# Music engagement, nutrition strategies, and psychological well-being in competitive athletes: a sequential explanatory mixed-methods study

**DOI:** 10.3389/fnut.2026.1934748

**Published:** 2026-10-05

**Authors:** Fangkun Li, Weiqiang Zhang

**Affiliations:** Department of P.E., Central South University, Changsha, Hunan, China

**Keywords:** athlete self-management, competitive athletes, mixed-methods, nutrition strategies, psychological well-being, recovery-related mental state, strategic music use, structural equation modeling

## Abstract

Competitive athletes routinely face psychological demands that intersect with daily behavioral practices, yet music engagement and nutrition strategies have rarely been examined together as correlates of athlete psychological well-being. This study investigated the cross-sectional associations of strategic music use and differentiated nutrition strategies with a psychological well-being and recovery-related mental state composite among competitive athletes, using a sequential explanatory mixed-methods design. Survey data were collected from 350 competitive athletes in Changsha, China, between February and May 2026, and semi-structured interviews were conducted with 30 purposively selected participants. Measurement validity was evaluated through exploratory and confirmatory factor analysis, and associations were estimated using multiple regression and structural equation modeling while adjusting for demographic and training covariates. Music engagement was positively associated with the psychological well-being composite (*β* = 0.31, 95% CI 0.22 to 0.41, *p* < 0.001), and nutrition strategies showed a comparably stronger association (*β* = 0.39, 95% CI 0.30 to 0.49, *p* < 0.001). Qualitative interviews indicated that athletes used music intentionally for arousal regulation, focus, and emotional recovery, while hydration and meal regularity were perceived as more feasible than diet planning, which was constrained by travel, scheduling, and resource barriers. These findings suggest that strategic music use and consistent nutrition practices represent behavioral correlates of athlete-reported psychological functioning and may inform non-clinical, athlete-centered self-management approaches, though longitudinal and intervention research is needed to evaluate directionality and causal mechanisms.

## 1 Introduction

Psychological well-being among competitive athletes has attracted sustained scientific and institutional attention over the past decade, driven by growing recognition that sport-related mental health concerns extend well beyond elite and professional settings (1). Prevalence data from systematic reviews and meta-analyses indicate that a substantial proportion of competitive athletes experience clinically relevant levels of anxiety, depressive symptoms, sleep disturbance, and emotional exhaustion at some point during their competitive careers, with reported rates of anxiety symptoms reaching 34% and depressive symptom prevalence ranging between 17 and 33% depending on the assessment instrument and competitive level examined (2). These figures are particularly notable because they challenge the long-standing assumption that sport participation is inherently protective, and they have motivated international sporting bodies including the International Olympic Committee (IOC) and the Federation of European Sport Psychology (FEPSAC) to develop consensus frameworks for athlete mental health monitoring, prevention, and early support (3). Against this backdrop, researchers have increasingly turned their attention to identifying naturally occurring athlete-initiated behavioral practices that are associated with more favorable psychological functioning, so that non-clinical self-management resources can be identified and made available to athletes across competitive levels and sport types (4).

Two behavioral domains that have independently attracted theoretical and empirical attention are music engagement and nutrition strategies, both of which are routinely embedded in competitive sport routines (5). In sport and exercise contexts, music use is recognized as a multidimensional psychological strategy capable of influencing arousal, motivation, emotional state, and attentional focus (6). Karageorghis and Priest established a taxonomy distinguishing synchronous music used to coordinate movement with tempo from asynchronous music used for emotional or motivational purposes before and after exercise and from dissociative music used to redirect attention away from physical sensations during sustained effort. Theoretical grounding for these effects has been sought in self-determination theory, which frames intrinsic motivation and autonomous regulation as drivers of psychological well-being, and in attentional resource models, which explain how music can occupy cognitive bandwidth and thus reduce awareness of effort or negative affect during demanding exercise (7). Nutrition occupies a distinct position as both a physiological performance requirement and a behavioral self-regulation demand. Sports nutrition frameworks emphasize energy availability, carbohydrate and protein periodization, hydration, and recovery nutrition as core components of athletic preparation (8), while nutritional psychiatry research has linked dietary quality and dietary improvement with reduced risk of depressive and anxiety-related outcomes in non-clinical populations (9). Hydration specifically has been shown to affect cognitive function, mood, and perceptions of fatigue even at mild deficit levels, making it relevant to the psychological as well as the physiological dimensions of athlete functioning.

The research literatures on music in sport and on sports nutrition have both grown considerably in depth yet they have largely developed in parallel without systematic integration (10). A comprehensive meta-analytic review by (11), synthesizing data across experimental and quasi-experimental designs found moderate positive effects of music on affective valence, motivation, and perceived exertion during exercise, with effect sizes varying as a function of music tempo, exercise intensity, and the degree to which music use was intentional rather than incidental (12). Studies examining pre-exercise and pre-competition music specifically have reported associations with elevated self-reported readiness, more positive pre-competition affect, and reduced competitive anxiety in athlete samples, suggesting that deliberate playlist selection for psychological preparation constitutes a distinct mode of engagement beyond mere background listening (13). On the nutrition side, cross-sectional research consistently demonstrates that athletes who report higher dietary quality, more consistent meal regularity, and adequate hydration also tend to report more favorable mood states, lower perceived stress, and better perceived recovery (14). Recovery-oriented nutrition practices encompassing post-exercise carbohydrate and protein intake timed to support muscle repair and glycogen replenishment have been associated with reduced overtraining symptoms and more favorable training readiness in athlete cohorts monitored prospectively (15). Collectively these bodies of evidence suggest that both music engagement and nutrition strategies represent behavioral correlates of athlete psychological functioning, yet no study has examined them simultaneously within a single athlete sample using a design capable of evaluating their relative and independent contributions (16).

A more granular examination of the existing literature reveals important distinctions that earlier integrated reviews have not adequately addressed. For music, the critical distinction lies between passive background listening and purposeful self-directed engagement (17). Studies that treat all music listening as equivalent behavioral exposure systematically underestimate the psychological specificity of intentional music use and conflate meaningfully different forms of engagement (18). Strategic music use defined as deliberate choices about timing, tempo, genre, and emotional valence in relation to sport demands is conceptually distinct from incidental or habitual listening and is more likely to reflect an active form of psychological self-regulation. For nutrition, the critical heterogeneity concerns the differential effort demands of distinct nutritional behaviors. Hydration and regular meal timing are relatively easy to embed within training routines through behavioral cues and team scheduling, whereas diet planning for travel, pre-competition fueling protocols, and recovery-oriented meal preparation require executive function, advance planning, financial resources, and adaptive scheduling, all of which may be particularly constrained in younger athletes competing at university or regional levels (19). This distinction between basic nutrition habits and complex nutrition planning has direct implications for understanding differential associations with psychological outcomes because effortful self-regulatory behaviors may confer different psychological correlates than routinized automatized habits (20). On the basis of this theoretical reasoning and available empirical evidence, the present study proposed that music engagement would be positively associated with the psychological well-being and recovery-related mental state composite, and that nutrition strategies particularly basic nutrition habits would show a comparably strong or stronger association, given that consistent daily fueling practices interact closely with fatigue, mood, and recovery perception in athlete functioning (21).

Despite this growing knowledge base, several important methodological and conceptual limitations continue to constrain the field. First, almost all existing research examining music effects in sport has used experimental or laboratory designs in which music is assigned rather than self-selected, which limits ecological validity for understanding how athletes naturally use music in their own preparation and recovery routines (22). Survey-based studies that would provide better ecological coverage have rarely distinguished between strategic and passive music use, and those that have done so have typically examined only one dimension of athlete well-being such as competitive anxiety or mood rather than a comprehensive psychological functioning composite. Second, nutrition research in athletic populations has predominantly focused on physiological outcomes including performance indices, body composition, and biochemical recovery markers, while psychological correlates of nutrition behaviors have received comparatively less systematic attention. The few studies examining nutrition and psychological well-being associations in athletes have typically used composite dietary quality scores without distinguishing between nutrition behaviors that differ substantially in their planning demands, effort intensity, and contextual feasibility (23). Third, mixed-methods designs combining quantitative association models with qualitative inquiry into athlete experiences have been notably rare in this field, meaning that quantitative patterns are frequently reported without contextual accounts of how athletes understand and enact these behaviors in their everyday training and competition lives. Fourth, studies that have examined behavioral predictors together have typically done so using simple regression without evaluating measurement quality through confirmatory factor analysis or addressing common method variance in self-report data, both of which are methodological requirements for credible latent-variable behavioral research (24).

The specific research gap addressed by the present study is the absence of an investigation that simultaneously examines music engagement and differentiated nutrition strategies as correlates of athlete psychological well-being within a single methodologically rigorous mixed-methods framework. Existing studies have examined these behavioral domains independently, have not distinguished between basic nutrition habits and complex nutrition planning, have rarely used validated latent-variable modeling to evaluate construct measurement quality, and have not combined quantitative association evidence with qualitative interview accounts of athlete experience in an integrated sequential explanatory design. Sequential explanatory mixed-methods designs in which quantitative findings are generated first and qualitative inquiry is used to explain and contextualize those findings are particularly well-suited to this research problem because they allow statistical patterns to be interpreted alongside participant accounts of how, why, and under what circumstances behavioral practices are enacted or constrained (25). Without this combination it is impossible to evaluate whether observed quantitative associations between music or nutrition variables and psychological outcomes reflect meaningful behavioral patterns or artifacts of construct overlap or common method variance (26). The present study was therefore designed to address this gap by collecting survey data from a competitive athlete sample, evaluating measurement properties of the instruments, modeling cross-sectional associations with regression and structural equation modeling, and conducting semi-structured interviews to provide explanatory context for the quantitative patterns (27).

The overarching aim of this study was to examine the cross-sectional associations of strategic music engagement and differentiated nutrition strategies with a psychological well-being and recovery-related mental state composite among competitive athletes, and to use qualitative interview accounts to explain how athletes experienced and made sense of these associations in everyday sport contexts. The specific objectives were as follows: first to evaluate the measurement properties of the study-specific music engagement and nutrition strategy instruments through exploratory and confirmatory factor analysis; second to estimate the associations of music engagement and overall nutrition strategies with the psychological well-being and recovery-related mental state composite while adjusting for demographic and training covariates using multiple regression and structural equation modeling; third to test whether basic nutrition habits and complex nutrition planning showed differential association magnitudes with the psychological outcome and to interpret any observed differences in light of athlete interview accounts; and fourth to assess the robustness of the quantitative findings using common method variance diagnostics including a three-pronged strategy of Harman’s single-factor test, single-factor confirmatory factor analysis comparison, and a latent common method factor sensitivity analysis. These objectives collectively required a sequential explanatory mixed-methods design in which quantitative analyses were conducted first and informed the purposive selection of interview participants and the development of the qualitative interview guide.

The present study contributes to the literature in several theoretically and methodologically distinct ways. It is among the first to examine music engagement and nutrition strategies simultaneously as correlates of athlete psychological well-being within a single well-powered competitive athlete sample, allowing their relative associations to be estimated and compared directly. By distinguishing between basic nutrition habits and complex nutrition planning the study addresses a conceptually important heterogeneity in the nutrition behavior literature that has practical implications for understanding which nutritional behaviors are more or less feasible for athletes at different competitive levels and under different training and travel demands. The use of exploratory and confirmatory factor analysis to evaluate instrument measurement quality combined with a three-pronged common method variance diagnostic strategy represents a methodological standard that is frequently absent from existing surveys of athlete behavioral practices and psychological outcomes. The sequential explanatory mixed-methods integration provides a richer interpretive framework than purely quantitative designs, enabling the study to situate statistical associations within athlete-reported experiences of how music and nutrition practices function in competitive sport routines. Taken together these contributions advance the empirical and methodological foundation for athlete-centered psychological self-management research and offer practically relevant insights for sport psychologists, coaches, and sports nutrition professionals seeking to support athlete well-being through feasible non-clinical behavioral strategies.

## 2 Materials and methods

### 2.1 Study design

This investigation employed a sequential explanatory mixed-methods design (QUAN → qual), in which a quantitative survey phase was conducted first, analyzed, and then used to inform and guide a subsequent qualitative interview phase (28). The design was selected because quantitative association models can identify the magnitude and direction of statistical relationships between behavioral practices and psychological outcomes, while qualitative inquiry provides contextual accounts that explain how and why those associations exist from the perspective of athletes who enact these practices within the constraints of competitive sport environments. The two phases were integrated at four planned points: purposive interview sampling was informed by quantitative results; the qualitative interview guide was developed to explore specific quantitative patterns particularly the differential scores observed for hydration and diet planning within the nutrition strategy measure; qualitative themes were compared with quantitative patterns through a joint display; and the discussion synthesizes both strands into an integrated interpretation. The study was observational and non-interventional throughout. No music, nutrition, therapy, training, or control condition was assigned or manipulated and no causal inference is claimed from the quantitative association models. Observational reporting was aligned with the Strengthening the Reporting of Observational Studies in Epidemiology (STROBE) statement, which specifies transparent reporting standards for participant characteristics, variable definitions, statistical procedures, and limitations in observational research (29).

### 2.2 Participants and sampling

Participants were recruited between February and May 2026 from competitive sport settings in Changsha, Hunan, China. Recruitment sites included universities with competitive sport programs, regional sports clubs, sports training centers, and regional sports organizations. A purposive convenience sampling approach was applied to identify adults aged 18 years or older who were actively involved in organized training and competition at university, regional, national, or professional levels. Athletes were excluded if they were not currently participating in competitive training at the time of data collection, did not complete the questionnaire to a sufficient degree to permit analysis, or did not meet minimum comprehension requirements for the survey instrument as indicated by response consistency checks. Of the 832 athletes initially approached, 520 agreed to participate, yielding a participation agreement rate of 62.5%. Of these, 412 completed and returned questionnaires, corresponding to a return rate of 79.2% among those who agreed to participate. After screening for response completeness and eligibility confirmation, 350 participants were retained for the final quantitative analysis. Of the 62 cases excluded after questionnaire return, 48 were removed due to incomplete item responses defined as more than 10% missing across scale items and 14 were removed because eligibility confirmation indicated they were not currently competing at the required competitive level at the time of data collection. The complete participant flow is depicted in Figure 1, which documents all recruitment stages, agreement rates, return rates, exclusion reasons, and final analytical samples for both the quantitative and qualitative phases.

**Figure 1:**
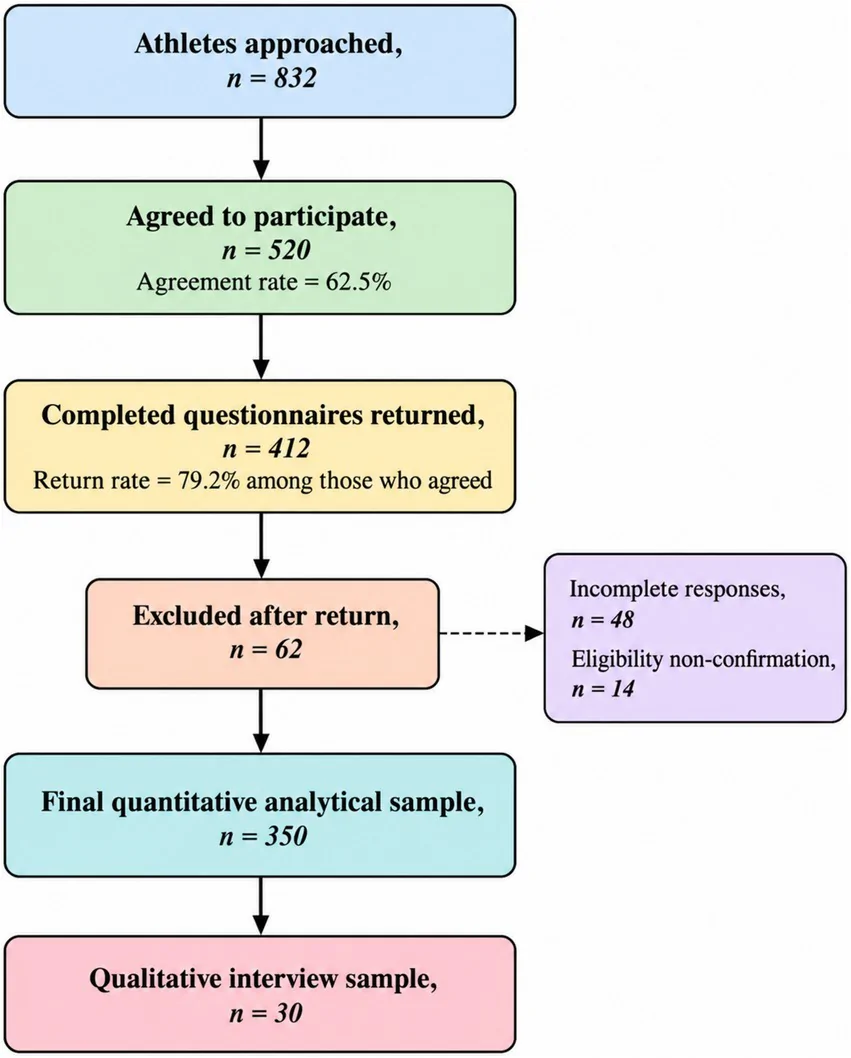
Flow diagram of participant recruitment and study sample. The diagram presents the number of athletes approached (*n* = 832), who agreed to participate (*n* = 520), who returned completed questionnaires (*n* = 412), who were excluded after return (*n* = 62; 48 for response incompleteness and 14 for eligibility non-confirmation), and who were included in the final quantitative analytical sample (*n* = 350) and the qualitative interview sample (*n* = 30).

### 2.3 Sample size justification

The required minimum sample size for the quantitative phase was estimated prospectively before data collection, separately for multiple regression and structural equation modeling. For the adjusted multiple regression model which included 9 predictors (2 behavioral predictors and 7 demographic and training covariates), a minimum sample size was calculated using *a priori* power analysis assuming a small-to-moderate effect size (*f*^2^ = 0.15), a two-tailed alpha level of 0.05, and a desired statistical power of 0.80. This procedure yielded a minimum required sample of approximately 114 participants. For the structural equation model, published guidelines recommend a minimum of 5 to 10 observations per estimated parameter for model stability, and the measurement model in the present study involved 21 freely estimated parameters across 4 latent constructs. These recommendations supported a minimum requirement of approximately 200 participants for the SEM component. The final quantitative sample of 350 participants comfortably exceeded both benchmarks, providing adequate statistical power for all planned analyses and supporting the stability of the measurement model.

### 2.4 Data collection procedure

Data collection proceeded in two sequential phases. In the first phase, athletes completed a structured self-report questionnaire encompassing demographic and sport-related information, music engagement items, nutrition strategy items, and the psychological well-being and recovery-related mental state composite items. Questionnaires were administered in two modes: 246 questionnaires (70.3% of the analyzable sample) were collected via an online survey platform and 104 (29.7%) were collected as paper-based mailed questionnaires. Identical item wording, response options, instructions, and scale anchors were used across both delivery modes. To evaluate whether questionnaire mode introduced systematic response differences, a sensitivity analysis was conducted comparing mean scores on all key constructs between online and paper-based respondents. No statistically significant differences were observed across any construct (all *p > 0.05*), supporting the equivalence of the two administration modes and permitting their combination in a single analytical dataset. Participants were fully informed of the study purpose, voluntary nature of participation, confidentiality of responses, and their right to withdraw at any stage without consequence.

In the second phase, 30 athletes were purposively selected from the quantitative sample for semi-structured qualitative interviews. Purposive selection was guided by the quantitative findings specifically the differential scores observed between hydration (*M* = 3.82) and diet planning (*M* = 3.28) within the nutrition strategy measure and by the aim of achieving diversity across sport type, sex, and competition level. Interviews were conducted by the first author who holds research training in sport psychology and mixed-methods inquiry and who received specific training in semi-structured interviewing, ethical research conduct, and participant-centered approaches before data collection began. The interviewer had no prior personal, clinical, or supervisory relationship with any participant. Each interview was conducted individually, lasted between 20 and 40 min, was audio-recorded with informed consent, and was conducted in Mandarin Chinese. Transcripts were prepared in Chinese and translated into English by a bilingual team member fluent in both languages, with discrepancies resolved through direct comparison of the original-language and translated versions. All participant quotations were anonymized before reporting, and coding and thematic organization were conducted manually using structured coding tables and a maintained audit trail.

### 2.5 Measures

#### 2.5.1 Development and content validation

The music engagement and nutrition strategy measures were developed specifically for this study because no existing instrument fully captured the behavioral specificity required to distinguish strategic from passive music use and basic nutrition habits from complex nutrition planning in competitive athlete populations. Item generation followed a theory-driven approach grounded in sport psychology frameworks for music use in exercise (30) and in sports nutrition position statements addressing hydration, energy availability, recovery nutrition, and competition-specific fueling (31). A preliminary pool of items was evaluated by a panel of experts with backgrounds in sport psychology, physical education, and sports dietetics who assessed item relevance, clarity, and content coverage. Items were refined based on expert feedback and pilot-tested with a convenience sample of 28 athletes to evaluate comprehensibility and preliminary response distributions. No major comprehension issues were identified, and pilot data supported the retention of all items for the main study. Because the resulting measures were substantially adapted from existing frameworks rather than adopted from a single previously validated questionnaire, all psychometric analyses are treated as preliminary evidence of internal structure and reliability within the present sample and further independent validation in diverse athlete populations is required.

#### 2.5.2 Demographic and sport-related covariates

Demographic and sport-related information was collected to characterize the sample and to serve as covariates in the regression and structural equation models. Variables collected included age (continuous, years), sex (male or female), sport type (team sport or individual sport), competition level (university, regional, or national and professional), training experience (continuous, years), weekly training frequency (sessions per week), and weekly training volume (hours per week). These variables were selected because previous research has identified associations between demographic and training characteristics and both music use patterns and nutritional behaviors in competitive athletes.

#### 2.5.3 Music engagement

Strategic music engagement in sport contexts was assessed using 8 structured self-report items. Each item was rated on a 5-point Likert scale anchored at 1 (strongly disagree) and 5 (strongly agree), with higher scores indicating greater strategic engagement with music during sport-related routines. The scale was designed to capture deliberate psychological uses of music for mental preparation, mood regulation, attentional focus, stress reduction, and emotional recovery rather than passive or incidental background listening. Table 1 presents the full item wording and the psychological domain targeted by each item.

**Table 1:** Music engagement items assessing athletes’ strategic use of music for psychological preparation, mood regulation, attentional focus, stress reduction, and emotional recovery in sport contexts.

Item code	Item wording	Purpose/domain
ME1	I use music before training to prepare myself mentally.	Strategic timing/mental preparation
ME2	I use music during warm-up or independent training to reach the right energy level.	Arousal regulation/energy management
ME3	I intentionally choose music to regulate my mood before training or competition.	Mood regulation/emotional control
ME4	I use music to improve focus or concentration during sport preparation.	Attentional focus/concentration
ME5	I use music to calm nervousness or stress before competition.	Stress reduction/anxiety management
ME6	I use music after training or competition to recover emotionally.	Emotional recovery/post-performance regulation
ME7	My sport-related music use is planned or strategic rather than only random background listening.	Strategic intentionality/deliberate use
ME8	I reflect on which playlists or songs help my competition mindset.	Strategic reflection/playlist planning

#### 2.5.4 Nutrition strategies

Nutrition strategies were assessed using 7 structured items rated on the same 5-point Likert scale. To address the conceptual heterogeneity of athlete nutrition behaviors, a two-subdimension structure was specified *a priori*. Basic nutrition habits comprised 4 items addressing behaviors that are relatively low in planning demand and that can be embedded into existing training routines: regular meal timing (NS1), adequate hydration (NS2), post-training recovery intake (NS4), and seasonal dietary quality maintenance (NS5). Complex nutrition planning comprised 3 items addressing behaviors requiring greater advance planning, resource allocation, and adaptive self-regulation: pre-training or pre-competition food planning (NS3), advance meal preparation to match training schedules (NS6), and food and fluid adjustment during travel or away competitions (NS7). This classification was motivated by theoretical reasoning that hydration and meal regularity involve different cognitive, motivational, and logistical demands than competition-specific diet planning, and by the expectation that these subdimensions might show different associations with psychological outcomes. The full item set is presented in Table 2.

**Table 2:** Nutrition strategy items assessing athletes’ basic nutrition habits and complex planning practices related to meals, hydration, recovery nutrition, diet quality, and competition travel.

Item code	Item wording	Sub-dimension
NS1	I maintain regular meals during training periods.	Basic nutrition habit
NS2	I maintain adequate hydration before, during, or after training.	Basic nutrition habit
NS3	I plan what to eat before training or competition.	Complex nutrition planning
NS4	I use post-training food or drink to support recovery.	Basic nutrition habit
NS5	I try to maintain an overall high-quality diet during the sport season.	Basic nutrition habit
NS6	I prepare or plan meals in advance to match my training schedule.	Complex nutrition planning
NS7	I adjust my food and fluid intake when travelling or competing away from home.	Complex nutrition planning

#### 2.5.5 Psychological well-being and recovery-related mental state composite

The primary outcome was conceptualized as a composite indicator of athlete-reported psychological functioning rather than a clinical assessment of mental health disorder. The composite was developed to reflect the multidimensional nature of psychological well-being in competitive sport, incorporating perceived stress, positive psychological well-being, mood state, fatigue, psychological readiness, and recovery perception. These six domains were selected because athlete well-being frameworks and recovery monitoring literatures consistently identify them as interrelated components of the psychological state experienced by athletes within daily training and competition cycles (32). Items within each domain were rated on a 5-point Likert scale and items assessing unfavorable states were reverse-coded before scoring so that higher scores uniformly indicated more favorable psychological functioning.

Domain scores were computed by averaging items within each domain after reverse coding. To prevent domains assessed by different numbers of items from contributing unequally to the composite, each domain score was z-score standardized before aggregation. The final composite score was calculated as the arithmetic mean of the six standardized domain scores, as expressed in Equation 1.

$$\text{PWBRMS}=\frac{1}{6}\sum_{d=1}^{6}z_{d}$$ (1)

In Equation 1, PWBRMS is the psychological well-being and recovery-related mental state composite score, d indexes the six domains (perceived stress, psychological well-being, mood, fatigue, readiness, and recovery perception), and z is the standardized score for domain d. Higher composite scores indicate more favorable self-reported psychological functioning. The domain structure, example item wording, and scoring direction for each domain are summarized in Table 3.

**Table 3:** Domain structure, construct assessed, example item wording, and scoring direction for the psychological well-being and recovery-related mental state composite.

Domain	Construct assessed	Example item	Scoring direction
Perceived stress	Current psychological strain during sport participation	“I feel able to manage stress related to training and competition.”	Higher score = lower perceived stress (reverse-coded items)
Psychological well-being	Positive psychological functioning	“I feel positive and mentally balanced during my sport participation.”	Higher score = better well-being
Mood	Current emotional state during sport participation	“I generally experience a positive mood during my training period.”	Higher score = more favorable mood state
Fatigue	Perceived tiredness related to training demands	“I feel excessively tired because of my training demands.”	Reverse-coded; higher score = lower fatigue burden
Readiness	Psychological preparedness for training and competition	“I feel mentally prepared before important training sessions or competitions.”	Higher score = greater perceived readiness
Recovery perception	Subjective adequacy of recovery	“I feel recovered and ready for upcoming training demands.”	Higher score = better perceived recovery

Because fatigue, readiness, and recovery perception also reflect physical condition and training status, the composite should not be interpreted as an equivalent to clinical mental health assessment. Potential conceptual overlap between predictor items, which included emotional regulation and recovery-related content, and the outcome composite, which included mood, stress, and recovery perception domains, was acknowledged explicitly during interpretation. All associations were described as relationships between related behavioral practices and psychological functioning rather than as evidence of independent causal pathways.

### 2.6 Measurement validation and quantitative data analysis

All statistical analyses were performed using IBM SPSS Statistics version 29.0 for exploratory analyses and R version 4.4.2 with the lavaan package (version 0.6–19) for confirmatory factor analysis, structural equation modeling, and common method variance diagnostics. Because the music engagement and nutrition strategy instruments were developed specifically for this study, all psychometric analyses were treated as preliminary evaluations of internal structure and measurement adequacy within the present sample rather than as definitive validation of the instruments.

Exploratory factor analysis (EFA) was conducted first to examine the underlying factor structure of the study items. The EFA was performed on 21 variables: the 8 music engagement items, the 7 nutrition strategy items, and the 6 domain-level scores representing the psychological well-being and recovery-related mental state composite (perceived stress, psychological well-being, mood, fatigue, readiness, and recovery perception). Factorability of the correlation matrix was assessed using the Kaiser–Meyer–Olkin (KMO) measure of sampling adequacy and Bartlett’s test of sphericity. For 21 variables the Bartlett degrees of freedom equal k(k − 1)/2 = 21 × 20/2 = 210. Factors were extracted using principal axis factoring with Promax oblique rotation because theoretical correlations between behavioral constructs were anticipated and orthogonal rotation would impose an unrealistic constraint. The number of factors to retain was determined using multiple convergent criteria: eigenvalues greater than 1.0, visual inspection of the scree plot, parallel analysis based on 1,000 random permutations of the observed data matrix, and theoretical interpretability of the resulting factor structure. Factors were retained only when they demonstrated conceptual consistency with the predefined constructs of music engagement, basic nutrition habits, complex nutrition planning, and psychological well-being and recovery-related mental state. The full item-by-item Promax-rotated PAF pattern matrix from the EFA, showing primary loadings and cross-loadings for all 21 variables across all four extracted factors, is presented in Table 4. The EFA loading values in Table 5 are pattern coefficients from SPSS principal axis factoring and are not interchangeable with the standardized CFA loadings reported in Table 6, as the two analyses use different estimation frameworks and serve different inferential purposes.

**Table 4:** Integrated joint display combining quantitative patterns, qualitative contextual accounts, and mixed-methods interpretation.

Quantitative finding	Qualitative contextual account	Representative quotation	Mixed-methods interpretation
Music engagement: *r* = 0.45, *β* = 0.31, *p* < 0.001	Athletes described deliberate playlist selection for arousal regulation, motivational focus, and emotional recovery before training and competition	“Before competition, I listen to the same playlist because it helps me calm down and focus. It is not therapy, but it helps me control my mood and prepare mentally.” (Participant 07, team sport)	Music engagement reflects active self-regulation rather than passive background listening
Hydration *M* = 3.82 > diet planning *M* = 3.28	Hydration embedded into training through portable bottles and team cues; diet planning constrained by travel, finance, and scheduling demands	“Drinking water is easy because I keep a bottle with me. Planning meals is harder—when we travel or have late practice, I eat whatever is available.” (Participant 14, regional)	Behavioral feasibility drives within-construct variability; basic habits and planning demand different support strategies
Nutrition strategies: *r* = 0.50, *β* = 0.39, *p* < 0.001	Athletes linked consistent intake and hydration with energy stability, reduced fatigue, and better psychological readiness	“When I eat regularly and stay hydrated, I feel less tired and less irritated. If I skip meals, my mood changes and I cannot concentrate.” (Participant 21, individual sport)	Nutrition practices support psychological functioning through physiological stability and perceived recovery
Music–nutrition correlation: *r* = 0.36, *p* < 0.001	Athletes integrated music, nutrition, sleep, and training as interconnected self-management systems	“Music, food, sleep, and training are all connected. If one part is missing, I feel less ready, so I keep the same routine before important sessions.” (Participant 03, national/professional)	Both practices may reflect a broader dispositional tendency toward deliberate athlete self-management

**Table 5:** Promax-rotated principal axis factoring (PAF) pattern matrix from exploratory factor analysis of all 21 variables (8 music engagement items, 7 nutrition strategy items, and 6 psychological well-being and recovery-related mental state domain scores).

Item	Factor 1 ME	Factor 2 BNH	Factor 3 CNP	Factor 4 PWBRMS
ME1—music before training (mental preparation)	0.79	0.09	0.06	0.11
ME2—music during warm-up (energy regulation)	0.76	0.12	0.08	0.09
ME3—intentional mood regulation before training	0.81	0.08	0.04	0.14
ME4—music for focus and concentration	0.75	0.11	0.07	0.10
ME5—music to calm nervousness before competition	0.68	0.07	0.10	0.18
ME6—music for emotional recovery after training	0.72	0.10	0.05	0.15
ME7—Strategic/planned music use	0.83	0.06	0.09	0.08
ME8—playlist reflection for competition mindset	0.80	0.08	0.11	0.06
NS1—regular meals during training	0.11	0.74	0.18	0.12
NS2—adequate hydration (before/during/after training)	0.08	0.78	0.10	0.16
NS4—post-training recovery intake	0.10	0.71	0.22	0.13
NS5—high-quality diet during sport season	0.13	0.69	0.19	0.14
NS3—pre-training/competition food planning	0.09	0.21	0.72	0.10
NS6—advance meal preparation for training schedule	0.07	0.19	0.76	0.08
NS7—food/fluid adjustment during travel	0.11	0.16	0.71	0.09
PWBRMS-D1—perceived stress (domain score)	0.13	0.14	0.08	0.73
PWBRMS-D2—psychological well-being (domain score)	0.16	0.17	0.09	0.80
PWBRMS-D3—mood (domain score)	0.14	0.15	0.07	0.78
PWBRMS-D4—fatigue (domain score)	0.10	0.18	0.11	0.74
PWBRMS-D5—readiness (domain score)	0.12	0.16	0.10	0.77
PWBRMS-D6—recovery perception (domain score)	0.11	0.20	0.12	0.76

ME = music engagement; BNH = basic nutrition habits; CNP = complex nutrition planning; PWBRMS = psychological well-being and recovery-related mental state. Factor extraction used principal axis factoring with Promax oblique rotation (delta = 0, kappa = 4). KMO = 0.87; Bartlett’s test: *χ*^2^ = 3246.58, df = 210, *p* < 0.001. Four factors were retained on the basis of parallel analysis, eigenvalues > 1.0, scree-plot inspection, and theoretical interpretability. No primary loading fell below 0.68 and no cross-loading exceeded 0.22, indicating high item-level factor purity across all 21 variables. These Promax pattern coefficients are from the EFA conducted in SPSS version 29.0 and are not interchangeable with the CFA standardized loadings in Table 6, which were obtained from a separate lavaan WLSMV estimation.

Primary loadings are shown in bold.

**Table 6:** Measurement validation results for study-specific constructs derived from the confirmatory factor analysis (CFA).

Construct	Items	CFA loading range	*α*	CR	AVE	Max HTMT	Latent factor correlations
Music engagement (ME)	8	0.64–0.83	0.87	0.89	0.51	0.52	ME↔BNH = 0.30; ME↔CNP = 0.22; ME↔PWBRMS = 0.45
Basic nutrition habits (BNH)	4	0.66–0.81	0.82	0.84	0.57	0.66	BNH↔CNP = 0.61; BNH↔PWBRMS = 0.50
Complex nutrition planning (CNP)	3	0.63–0.78	0.78	0.80	0.53	0.62	CNP↔PWBRMS = 0.38
Psychological well-being and recovery-related mental state (PWBRMS)	6	0.67–0.85	0.88	0.90	0.60	0.66	Reference construct

All standardized factor loadings exceeded 0.60. All HTMT values were below the 0.85 threshold, supporting discriminant validity. Latent factor correlations are derived from the CFA model estimated in lavaan (version 0.6–19). ME = music engagement; BNH = basic nutrition habits; CNP = complex nutrition planning; PWBRMS = psychological well-being and recovery-related mental state. The column heading “CFA Loading Range” reflects that these values are standardized loadings from the confirmatory factor analysis and are not interchangeable with the EFA Promax pattern coefficients. The complete item-by-item EFA loading matrix is presented in Table 5.

α = Cronbach’s alpha; CR = composite reliability; AVE = average variance extracted; HTMT = heterotrait-monotrait ratio. Latent factor correlations are reported in the final column.

Confirmatory factor analysis (CFA) was subsequently performed in lavaan to evaluate the hypothesized four-factor measurement model. Because all items were measured using 5-point Likert scales with ordinal response categories, estimation used the weighted least squares mean and variance adjusted (WLSMV) estimator, which is appropriate for ordinal observed variables and does not assume multivariate normality. Factor loadings were constrained to their theoretical constructs and covariances between all pairs of latent constructs were freely estimated. Items were retained when they demonstrated standardized factor loadings of 0.50 or above, contributed meaningfully to construct reliability, and showed no substantial cross-loadings. Because EFA and CFA were both conducted in the same sample of 350 athletes due to the absence of a sufficiently large independent holdout sample, the resulting psychometric evidence is explicitly treated as preliminary and sample-specific and replication in independent athlete samples is required before the instruments can be considered fully validated.

Internal consistency was quantified using Cronbach’s alpha (α) for each construct. Composite reliability (CR) was calculated using Equation 2 to provide a reliability estimate less sensitive to the number of items than Cronbach’s alpha.

$$\mathrm{CR}=\frac{{\left(\sum \lambda_{i}\right)}^{2}}{{\left(\sum \lambda_{i}\right)}^{2}+\sum \delta_{i}}$$ (2)

In Equation 2, λᵢ is the standardized factor loading for item i and δᵢ = 1 − λᵢ^2^ is the corresponding item error variance. Convergent validity was evaluated using the average variance extracted (AVE), computed using Equation 3.

$$\mathrm{AVE}=\frac{\sum {\lambda_{i}}^{2}}{n}$$ (3)

In Equation 3, n is the number of items in the construct. An AVE value of 0.50 or above was used as the threshold for acceptable convergent validity. Discriminant validity was evaluated using the heterotrait-monotrait (HTMT) ratio of correlations with a threshold of 0.85. Latent factor correlations from the CFA were also reported separately from HTMT values to provide an additional perspective on construct distinctiveness. The measurement validation results are presented in Table 6.

Missing data were addressed before analysis. The complete-case (listwise) approach was applied, resulting in the exclusion of 48 questionnaires with more than 10% item-level missingness. The final analytical sample of 350 participants contained no missing values on any study variable. The robustness of this approach was evaluated through a sensitivity analysis comparing the complete-case regression estimates with those obtained under multiple imputation by chained equations (MICE with 20 imputed datasets). Regression coefficients varied by less than 5% between approaches and the direction and statistical significance of all main associations were unchanged, supporting the robustness of the complete-case findings.

### 2.7 Common method variance diagnostics

Because all quantitative constructs were assessed through self-report questionnaires administered at a single time point, common method variance (CMV) was considered a potential source of bias. Three complementary diagnostic approaches were applied following established recommendations for CMV assessment in behavioral survey research (33). First, Harman’s single-factor test was used as a preliminary screen, with the criterion that a single unrotated factor explaining more than 50% of total variance would be considered a warning sign warranting further investigation. Second, a one-factor CFA model was specified in lavaan and compared with the hypothesized four-factor measurement model; a substantially better fit of the multi-factor model relative to the single-factor model indicates that the observed relationships are not fully attributable to a single shared method factor. Third, a latent common method factor (LCMF) sensitivity analysis was conducted by adding an orthogonal unmeasured method factor to the hypothesized CFA model with all observed indicators allowed to load simultaneously on their substantive latent construct and on the method factor. The variance of the common method factor was fixed to 1.0 for model identification and all method-factor loadings were freely estimated. Specifically, all 21 indicators entered the LCMF model (8 music engagement items, 7 nutrition strategy items, and 6 PWBRMS domain scores), yielding 21 freely estimated method-factor loadings alongside the substantive factor loadings; fixing the method-factor variance to 1.0 provided the single identification constraint required to render the model just-identified with respect to the method factor. The proportion of method-related variance was estimated as the average squared standardized method-factor loading across indicators, and structural path estimates from the model with and without the method factor were compared to assess the practical sensitivity of the main findings to CMV. The CMV diagnostic results are presented in Table 7 and Table 8.

**Table 7:** Common method variance diagnostic results from three complementary approaches.

Diagnostic approach	Result	Decision criterion	Interpretation
Harman’s single-factor test	First unrotated factor explained 29.84% of total variance	Single factor > 50% of variance warrants concern	No dominant single factor; CMV is not the primary explanation for the observed associations
Single-factor CFA model comparison	*χ*^2^/df = 6.37, CFI = 0.614, TLI = 0.572, RMSEA = 0.124 (single-factor) versus *χ*^2^/df = 1.59, CFI = 0.952, TLI = 0.943, RMSEA = 0.051 (hypothesized model)	Markedly better multi-factor fit supports construct separation	Multi-factor model fit is substantially superior; construct distinctiveness is supported
Latent common method factor (LCMF) sensitivity analysis	Structural paths changed by 0.01–0.04; average method-related variance = 11.6%; LCMF model fit: *χ*^2^(223) = 341.76, CFI = 0.957, TLI = 0.949, RMSEA = 0.048, SRMR = 0.041	Path changes > 0.10 or method variance > 25% indicate substantial CMV	Main associations were robust to the inclusion of a method factor; residual CMV influence cannot be fully excluded

**Table 8:** Model fit statistics for the hypothesized CFA model and the CFA model augmented with a latent common method factor (LCMF).

Model	*χ* ^2^	df	*χ*^2^/df	CFI	TLI	RMSEA	SRMR
Hypothesized CFA model	356.42	224	1.59	0.952	0.943	0.051	0.045
CFA + latent common method factor	341.76	223	1.53	0.957	0.949	0.048	0.041

CFI = comparative fit index; TLI = Tucker–Lewis index; RMSEA = root mean square error of approximation; SRMR = standardized root mean square residual. The LCMF was specified orthogonal to all substantive latent constructs, with variance fixed to 1.0 and all method-factor loadings freely estimated. The marginal improvement in fit and the stability of structural coefficients (change ≤ 0.04) indicate that common method variance is unlikely to account for the principal findings.

### 2.8 Structural association models

Cross-sectional associations between music engagement, nutrition strategy dimensions, and the psychological well-being and recovery-related mental state composite were estimated using two complementary analytical approaches. Multiple regression was conducted using the observed composite score as the dependent variable allowing the incremental associations of each behavioral predictor to be estimated while controlling for age, sex, sport type, competition level, training experience, and weekly training volume simultaneously. Standardized regression coefficients (*β*), standard errors, 95% confidence intervals, and two-tailed *p*-values are reported. Statistical significance was evaluated at *α* = 0.05.

Structural equation modeling was conducted in lavaan using the WLSMV estimator. The SEM consisted of a measurement model component in which latent constructs were defined by their observed indicators and a structural model component in which directional paths were specified from music engagement and the nutrition strategy dimensions to the psychological well-being and recovery-related mental state latent variable. Demographic and training covariates were included as observed variables with direct paths to the outcome latent variable. Predictor latent constructs were allowed to covary freely because music engagement and nutrition behaviors were expected to reflect a partially shared tendency toward athlete self-management. A schematic representation of the structural model is presented in Figure 2.

**Figure 2:**
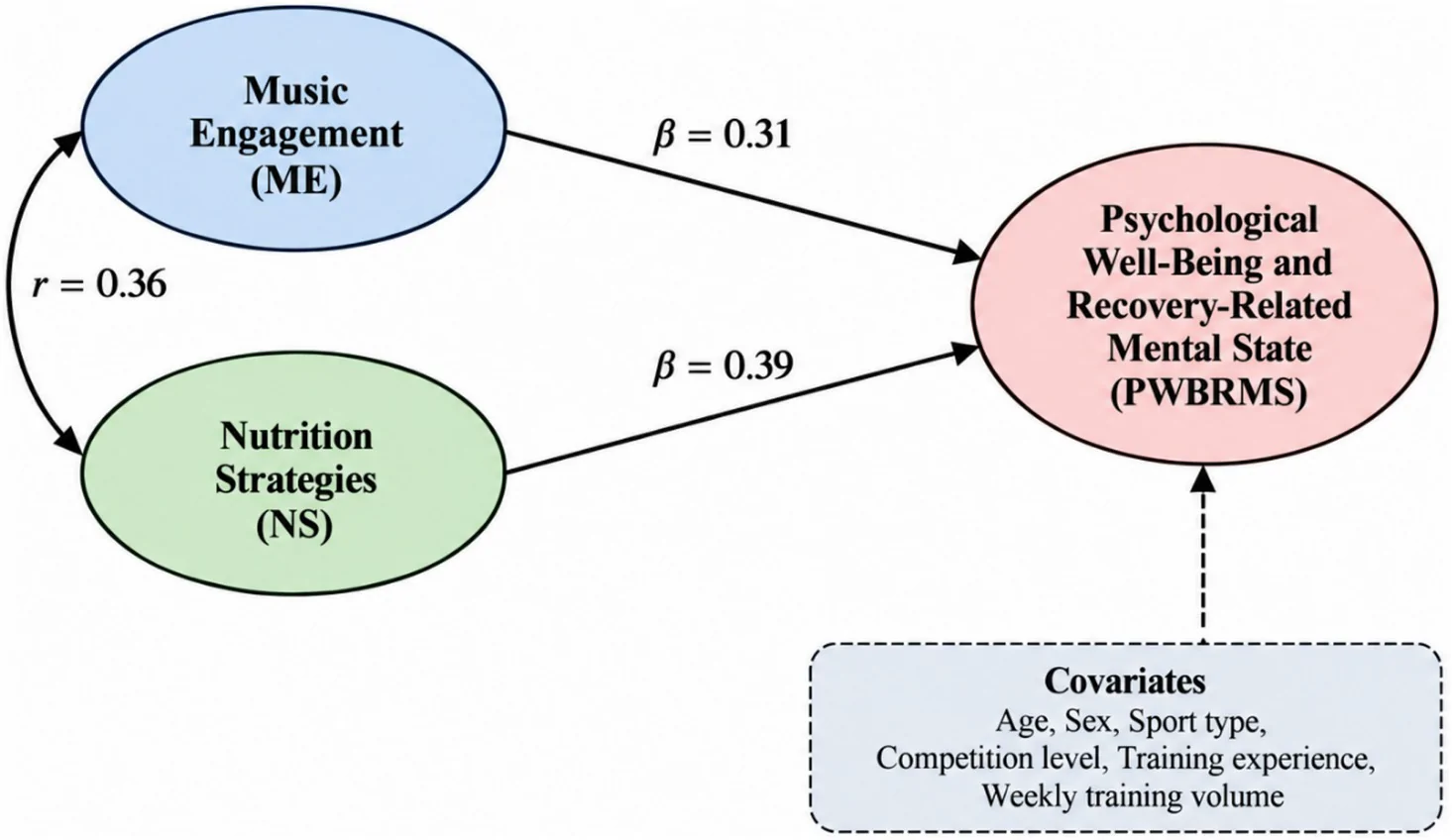
Structural equation model of associations among music engagement, nutrition strategies, and psychological well-being and recovery-related mental state. Standardized path coefficients are shown for the main structural associations. Single-headed arrows represent directional structural paths and double-headed arrows represent covariances. All associations are interpreted as cross-sectional statistical relationships and not as evidence of causal direction.

SEM model fit was evaluated using *χ*^2^ with its degrees of freedom, the *χ*^2^/df ratio, the comparative fit index (CFI), the Tucker–Lewis index (TLI), the root mean square error of approximation (RMSEA) with its 90% confidence interval, and the standardized root mean square residual (SRMR). Acceptable fit was defined as *χ*^2^/df ≤ 3.0, CFI and TLI ≥ 0.90, RMSEA ≤ 0.08, and SRMR ≤ 0.08, with excellent fit indicated by CFI and TLI ≥ 0.95 and RMSEA ≤ 0.05. All SEM results including standardized path coefficients with standard errors and 95% confidence intervals are reported in Section 3. All associations were described using observational language and directional arrows in the SEM were treated as theory-guided statistical specifications rather than evidence of causal direction Figure 3.

**Figure 3:**
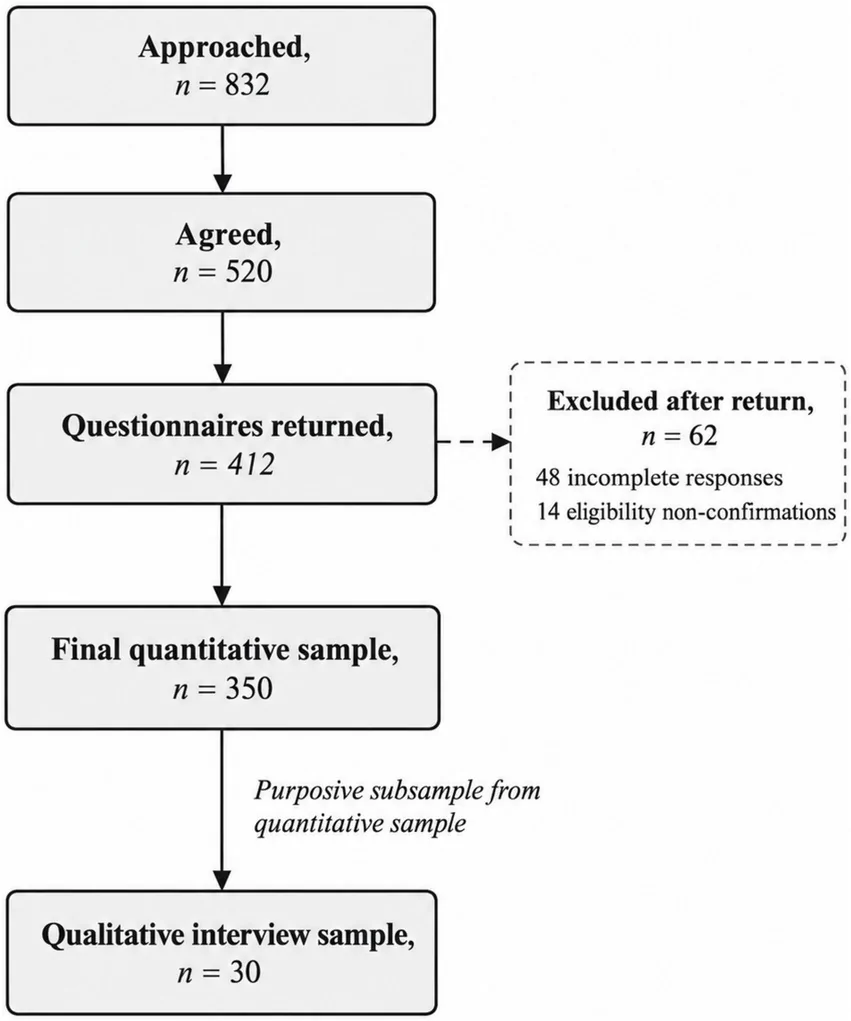
Flow diagram of participant recruitment and study sample for the quantitative survey (*n* = 350) and qualitative interview (*n* = 30) phases. The diagram presents the number approached (*n* = 832), who agreed (*n* = 520), who returned questionnaires (*n* = 412), reasons for exclusion, and final analytical samples.

### 2.9 Qualitative data analysis

Qualitative interview data were analyzed using codebook thematic analysis within a critical-realist epistemological orientation. This approach was selected because it combines the transparency and replicability of a structured codebook with sensitivity to participants’ accounts as meaningful reports of experienced realities shaped by contextual, cultural, and structural factors (34). The analysis proceeded through five stages. The research team first familiarized themselves with all 30 interview transcripts through repeated reading and initial memoing. An initial codebook was then developed deductively from the quantitative results and interview guide incorporating codes for music use purposes, nutrition behavior patterns, feasibility and barrier themes, and self-management integration. Inductive codes were added iteratively when participants described experiences not captured by the initial codebook. The codebook was refined by clarifying inclusion and exclusion criteria for each code, which were then applied systematically across all transcripts by the primary coder. A second investigator independently coded 20% of the transcripts to assess coding consistency and discrepancies were resolved through discussion and codebook refinement. Related codes were progressively organized into candidate themes, which were reviewed against the full coded dataset and the complete transcripts before being finalized.

Thematic sufficiency was monitored throughout the coding process rather than applied as a formal stopping rule. After approximately 24 interviews had been coded, no major new explanatory codes were generated by subsequent transcripts; the remaining 6 interviews were therefore analyzed to confirm the applicability of developing themes rather than to extend the codebook. This process was treated as an indication of thematic sufficiency within the present sample and is not interpreted as a claim of theoretical saturation applicable beyond this study context. Reflexive notes maintained by the first author throughout data collection and analysis were used to consider how researcher assumptions and expectations may have influenced coding decisions and interpretive choices. The absence of a prior personal, clinical, or supervisory relationship between the interviewer and participants was intended to reduce social desirability in interview responses.

### 2.10 Integration of quantitative and qualitative findings

Integration of the quantitative and qualitative strands occurred at four planned points consistent with sequential explanatory mixed-methods integration principles (35). Quantitative results informed the purposive selection of interview participants to ensure coverage of the full range of behavioral scores and competitive levels. The interview guide was developed to probe specific quantitative patterns in particular the observation that hydration scored substantially higher than diet planning within the nutrition strategy measure and the positive associations between both behavioral predictors and the psychological outcome composite. Qualitative themes were aligned with quantitative findings through a structured joint display which organized statistical patterns alongside contextual accounts and representative participant quotations to facilitate systematic comparison and meta-inference. The integrated discussion synthesizes both strands to explain how and under what contextual conditions athletes engage in strategic music use and nutrition-related self-management and why certain behaviors appear more consistently associated with favorable psychological functioning than others.

### 2.11 Ethical considerations and clinical trial registration

The study was conducted in accordance with the principles of the Declaration of Helsinki and applicable institutional ethical requirements. Ethical approval was granted by the Ethics Committee of the Department of Physical Education at Central South University before any data collection commenced (approval number CSU-PE-2026-34-0349; approval date 3 January 2026). All participants received written information describing the study purpose, procedures, voluntary nature of participation, confidentiality assurances, and their unconditional right to withdraw at any stage without penalty. Written informed consent was obtained from every participant before survey completion and before any interview recording. The study constituted a minimal-risk observational investigation and participants could skip any question or withdraw from the interview at any time. All personal identifiers were removed from data before analysis and survey and interview data were used solely for academic research purposes. Because this study was observational and did not assign participants to any music, nutrition, therapeutic, or training intervention, clinical trial registration was not required under standard interventional criteria. All results are interpreted as cross-sectional associations rather than treatment or intervention effects.

## 3 Results

### 3.1 Participant characteristics and study flow

The final quantitative sample comprised 350 competitive athletes drawn from universities, sports clubs, and regional training centers in Changsha, Hunan, China, between February and May 2026. Table 9, presented below, summarizes the demographic and sport-related characteristics of both the quantitative and qualitative samples. The quantitative sample had a mean age of 22.85 years (SD = 3.66), was predominantly male (55.7%), and was distributed across team (58.0%) and individual (42.0%) sports. The largest competition-level group was university athletes (46.3%), followed by regional (29.7%) and national or professional athletes (24.0%). Mean training experience was approximately 7 years, with athletes training on average 5.3 sessions and 10.5 h per week. The mean music engagement score was 3.65 (SD = 0.69) and the mean nutrition strategy score was 3.49 (SD = 0.65) on 5-point scales, indicating moderate-to-high endorsement of both behavioral practices across the sample. The mean psychological well-being and recovery-related mental state (PWBRMS) composite score was 3.62 (SD = 0.59). These descriptive patterns indicate that both music engagement and nutrition strategies were commonly practiced self-management behaviors among participants, establishing the behavioral basis for the association analyses that follow.

**Table 9:** Demographic and sport-related characteristics of the quantitative survey sample (*n* = 350) and the qualitative interview sample (*n* = 30).

Variable	Category	Quantitative (*n* = 350)	Interview (*n* = 30)
Age (years)	Mean ± SD	22.85 ± 3.66	21.78 ± 2.72
Sex	Male	195 (55.7%)	20 (66.7%)
	Female	155 (44.3%)	10 (33.3%)
Sport type	Team sports	203 (58.0%)	18 (60.0%)
	Individual sports	147 (42.0%)	12 (40.0%)
Competition level	University	162 (46.3%)	10 (33.3%)
	Regional	104 (29.7%)	10 (33.3%)
	National/Professional	84 (24.0%)	10 (33.3%)
Training experience (years)	Mean ± SD	6.99 ± 4.04	5.63 ± 2.93
Weekly training (sessions/week)	Mean ± SD	5.33 ± 1.15	5.23 ± 1.10
Weekly training volume (hours/week)	Mean ± SD	10.48 ± 3.40	11.02 ± 3.27
Music engagement score (1–5)	Mean ± SD	3.65 ± 0.69	3.87 ± 0.61
Nutrition strategy score (1–5)	Mean ± SD	3.49 ± 0.65	3.70 ± 0.58
PWBRMS composite score	Mean ± SD	3.62 ± 0.59	3.66 ± 0.65

PWBRMS = psychological well-being and recovery-related mental state composite. Scores are on a 1–5 Likert scale unless otherwise noted.

### 3.2 Music engagement patterns

Figure 4, shown below, presents the proportion of athletes endorsing each purpose of music use, defined as a rating of 4 or 5 on the 5-point Likert scale. Music use before training was endorsed by 78% of participants, during warm-up by 72%, and before competition by 69%. The most frequently endorsed psychological purposes were motivation (81%), mood regulation (74%), attentional focus (68%), and stress control (57%). These figures carry a substantive implication: the majority of athletes did not use music as incidental background sound but as a deliberately chosen instrument of psychological preparation. The dominance of motivational and mood-regulatory purposes over stress control endorsement suggests a hierarchical pattern in which athletes prioritize energy enhancement and emotional priming over anxiety management during routine preparation. This distinction aligns with theoretical frameworks proposing that music functions primarily as a pre-performance arousal-regulation tool in non-clinical athlete populations, rather than as a coping mechanism for competitive anxiety, and it helps explain why the overall music engagement score (*M* = 3.65, SD = 0.69) reflects moderate-to-high intentional engagement across the sample.

**Figure 4:**
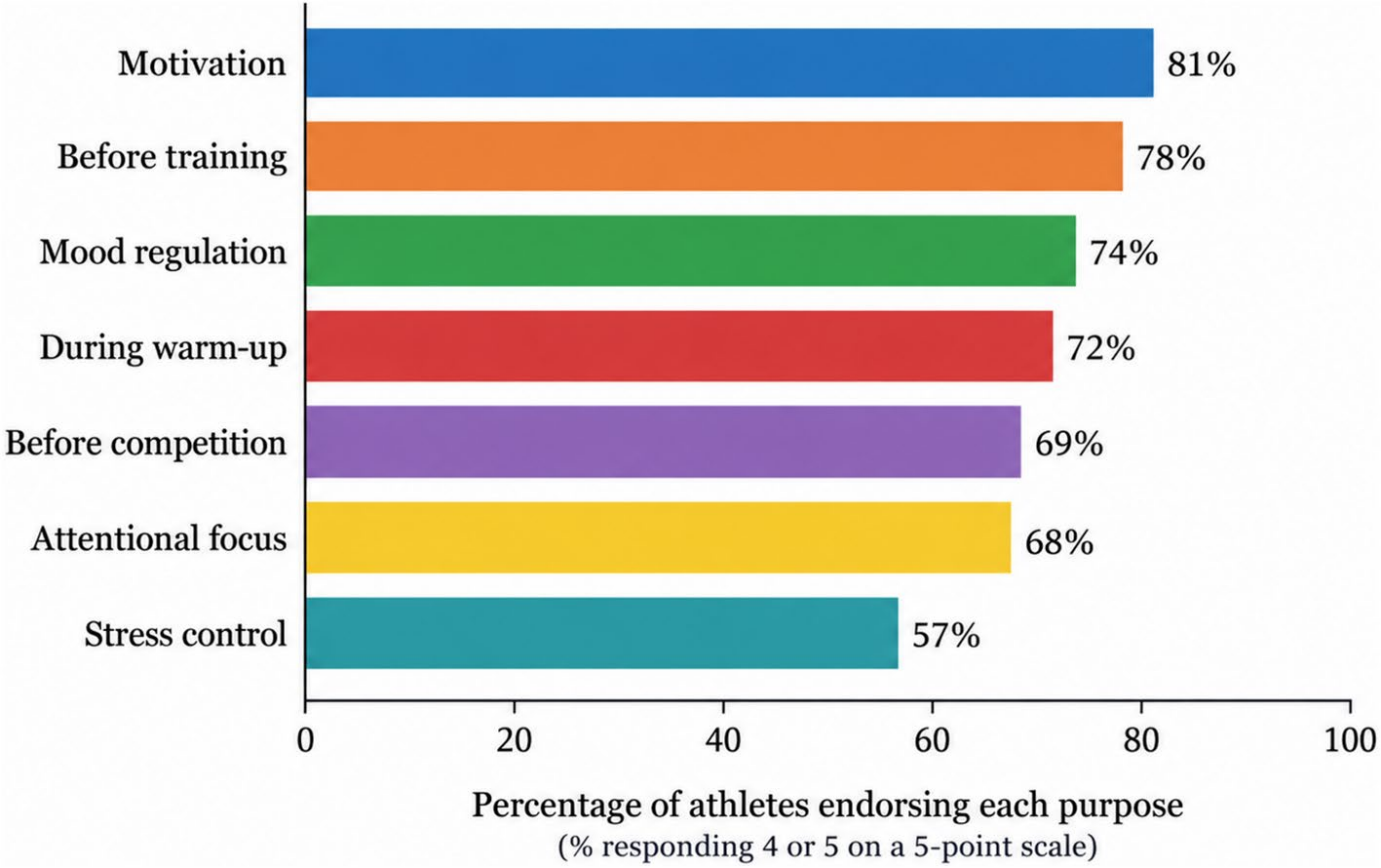
Proportions of athletes endorsing each music engagement purpose (% responding 4 or 5 on a 5-point scale). Purposes correspond directly to the music engagement items in Table 1. Bars are ordered from highest to lowest endorsement rate.

### 3.3 Nutrition strategy patterns

Mean scores across all seven nutrition strategy indicators are presented in Figure 5 below. Hydration received the highest endorsement (*M* = 3.82), followed by meal regularity (*M* = 3.61) and perceived dietary quality (*M* = 3.52). Recovery nutrition after training scored moderately (*M* = 3.44), while pre-competition food planning (*M* = 3.35) and travel nutrition adjustment (*M* = 3.31) scored lower. Diet planning received the lowest score of all indicators (*M* = 3.28). The substantive interpretation of this gradient is that behaviors requiring less cognitive planning and fewer logistical resources—particularly hydration and regular meal timing are more consistently practiced than behaviors demanding advance organization, financial resources, and scheduling flexibility. This finding has direct relevance for understanding athlete nutritional self-management: the within-construct variability signals that treating nutrition strategies as a single undifferentiated construct would mask meaningful behavioral heterogeneity, and that support targeting high-effort planning behaviors may require different intervention strategies than those targeting routine daily habits.

**Figure 5:**
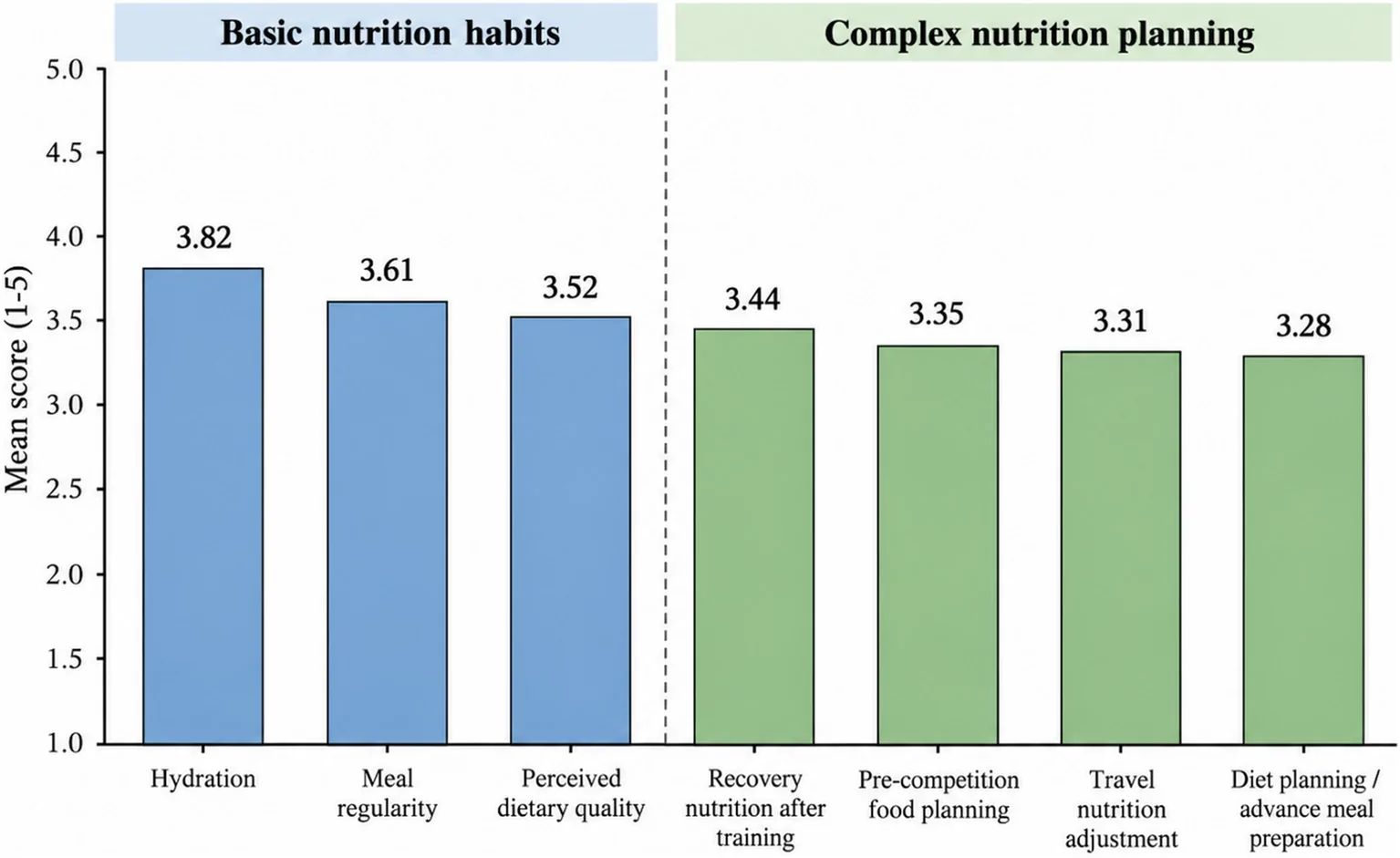
Mean scores for all seven nutrition strategy indicators on a 1–5 scale. Indicators are ordered from highest to lowest mean score. Basic nutrition habits (hydration, meal regularity, recovery intake, dietary quality) are displayed separately from complex nutrition planning behaviors (pre-competition planning, advance meal preparation, travel nutrition adjustment). Error bars represent ± 1 SD.

### 3.4 Measurement validity and common method variance diagnostics

Before estimating structural associations, the internal structure and measurement adequacy of the study instruments were evaluated through two sequential steps: an exploratory factor analysis (EFA) followed by a confirmatory factor analysis (CFA), as described in Section 2.6. The complete item-by-item Promax-rotated PAF pattern matrix from the EFA is presented in Table 5. Exploratory factor analysis supported the retention of four factors corresponding to the hypothesized constructs: music engagement, basic nutrition habits, complex nutrition planning, and the PWBRMS composite (36). The Kaiser–Meyer–Olkin measure was 0.87 and Bartlett’s test of sphericity was significant (*χ*^2^ = 3246.58, df = 210, *p* < 0.001), confirming adequate sampling and factorability of the correlation matrix. As shown in Table 5, all primary EFA loadings ranged from 0.68 to 0.83 across the 21 variables, and no cross-loading exceeded 0.22, indicating high item-level factor purity. Each of the 8 music engagement items loaded exclusively on Factor 1, the 4 basic nutrition habit items loaded exclusively on Factor 2, the 3 complex nutrition planning items loaded exclusively on Factor 3, and all 6 PWBRMS domain scores loaded exclusively on Factor 4, with negligible cross-factor associations throughout. This pattern provides empirical confirmation that the four constructs are distinct entities rather than expressions of a single underlying behavioral dimension, which is a prerequisite for the meaningful interpretation of separate structural paths in the SEM. The absence of substantial cross-loadings also rules out the possibility that the observed associations between music engagement, nutrition strategies, and PWBRMS are attributable to item content overlap between adjacent constructs. The loading ranges reported in Table 6 are from the subsequent CFA and are not interchangeable with the EFA pattern coefficients in Table 5, as stated in the Table 6 footnote. CFA-derived reliability and validity indices showed Cronbach’s α ranging from 0.78 to 0.88, composite reliability from 0.80 to 0.90, and average variance extracted from 0.51 to 0.60, collectively indicating satisfactory convergent validity and internal consistency for all constructs. The heterotrait-monotrait (HTMT) ratio did not exceed 0.66 for any construct pair (threshold 0.85), supporting discriminant validity. These measurement properties confirm that the constructs were sufficiently well-defined and distinct to support interpretable structural estimation (37).

Common method variance (CMV) was assessed through three complementary diagnostics. Harman’s single-factor test indicated that the first unrotated factor explained 29.84% of total variance, well below the 50% concern threshold. The single-factor CFA model showed substantially poorer fit (*χ*^2^/df = 6.37; CFI = 0.614; RMSEA = 0.124) than the hypothesized four-factor model (*χ*^2^/df = 1.59; CFI = 0.952; RMSEA = 0.051), confirming that the data are not attributable to a single shared method factor. The latent common method factor (LCMF) sensitivity analysis, summarized in Table 10, showed that adding an orthogonal method factor marginally improved fit while structural path estimates changed by only 0.01 to 0.04. Average method-related variance was 11.6%. Taken together these diagnostics indicate that CMV is unlikely to be the primary explanation for the observed associations, though residual self-report bias cannot be fully excluded given the single-occasion cross-sectional design.

**Table 10:** Model fit statistics for the hypothesized CFA model and the CFA model augmented with a latent common method factor (LCMF).

Model	*χ* ^2^	df	*χ*^2^/df	CFI	TLI	RMSEA	SRMR
Hypothesized CFA model	356.42	224	1.59	0.952	0.943	0.051	0.045
CFA + latent common method factor	341.76	223	1.53	0.957	0.949	0.048	0.041

CFI = comparative fit index; TLI = Tucker–Lewis index; RMSEA = root mean square error of approximation; SRMR = standardized root mean square residual. Marginal improvement in fit upon inclusion of the method factor, together with stable structural paths (change ≤ 0.04), indicates that common method variance does not account for the principal findings.

### 3.5 Correlations among study variables

Pearson correlations among the behavioral constructs and demographic characteristics are displayed in Figure 6. Music engagement was positively correlated with overall nutrition strategies (*r* = 0.36), basic nutrition habits (*r* = 0.30), and complex nutrition planning (*r* = 0.22). The moderately strong correlation between music engagement and nutrition strategies is behaviorally meaningful: it suggests that athletes who engage strategically with music also tend to maintain more structured nutritional routines, consistent with the qualitative theme of integrated self-management in which athletes described these practices as interdependent components of a coherent preparation system. Weekly training volume was modestly negatively associated with the PWBRMS composite (*r* = −0.12), indicating that heavier training loads correspond slightly to lower reported psychological well-being a pattern interpretable as accumulated training stress and consistent with the overtraining literature. Age and competition level showed negligible correlations with both behavioral predictors and the outcome, suggesting that the music-nutrition-wellbeing pattern is relatively stable across competitive stages represented in this sample.

**Figure 6:**
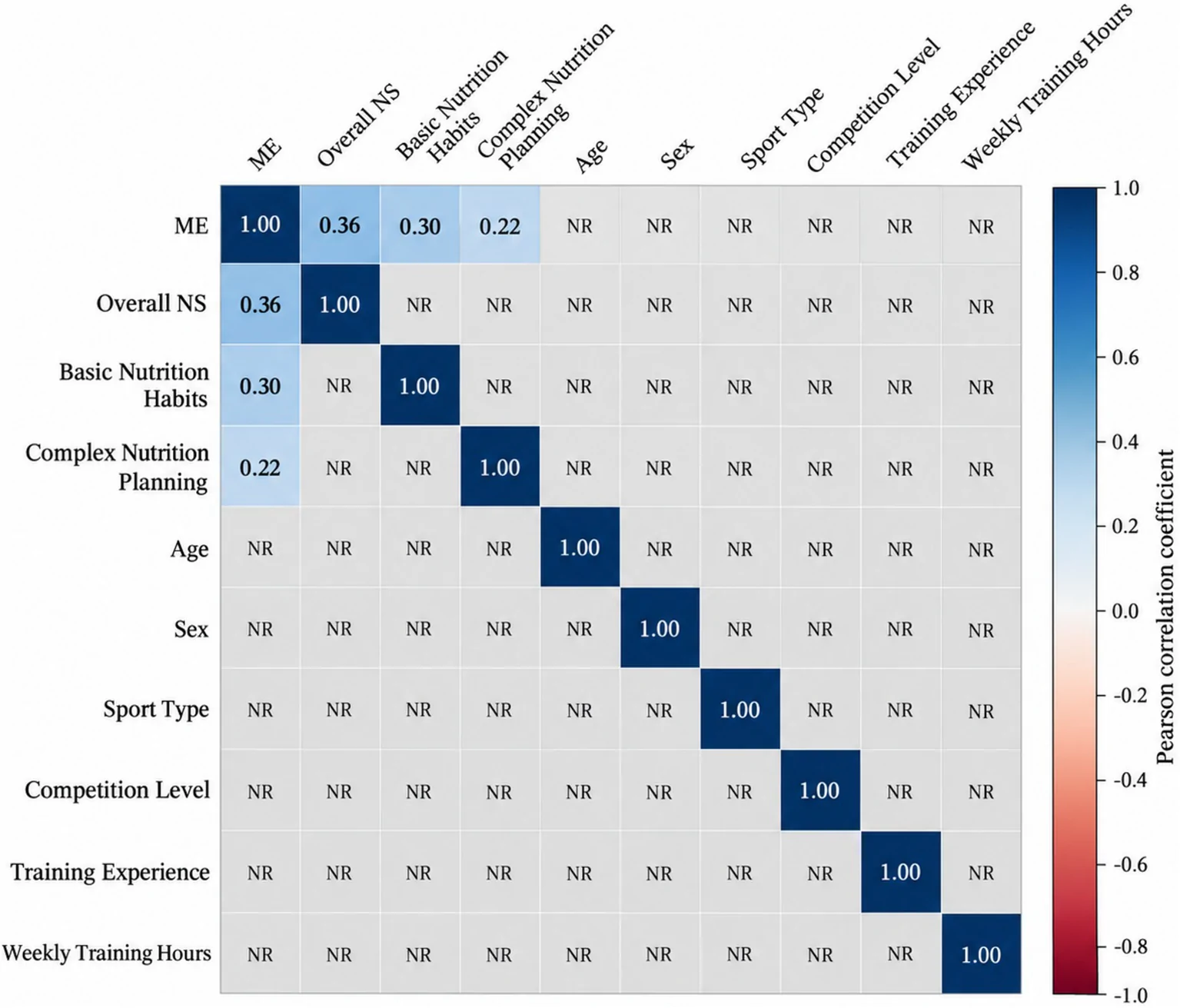
Pearson correlation matrix among music engagement, nutrition strategy dimensions, and demographic and training characteristics. Color intensity reflects the magnitude of the correlation coefficient. The PWBRMS composite and its domain scores were excluded from this matrix to avoid part-whole correlations with their component indicators.

### 3.6 Regression and SEM association models

Table 11 presents the adjusted regression coefficients (Panel A), SEM structural path estimates (Panel B), and SEM fit indices (Panel C). The SEM fit indices indicate excellent model fit: *χ*^2^/df = 1.84, CFI = 0.958, TLI = 0.949, RMSEA = 0.049 (90% CI: 0.039 to 0.058), and SRMR = 0.041. Figure 7 presents the full SEM path diagram with standardized coefficients.

**Table 11:** Adjusted regression (Panel A) and SEM structural model (Panel B) association results for music engagement and nutrition strategies with the psychological well-being and recovery-related mental state composite, with SEM model fit indices (Panel C).

Panel A. adjusted regression model	*β*	SE	95% CI	*P*
Age	0.06	0.09	−0.11 to 0.24	0.474
Sex (female vs. male)	0.02	0.04	−0.07 to 0.11	0.625
Individual vs. team sport	0.01	0.04	−0.08 to 0.10	0.791
Regional vs. university level	0.03	0.05	−0.06 to 0.12	0.538
National/professional vs. university level	−0.01	0.05	−0.10 to 0.09	0.909
Training experience	−0.06	0.09	−0.23 to 0.12	0.504
Weekly training hours	−0.09	0.04	−0.18 to −0.00	0.048
Music engagement → PWBRMS	0.31	0.05	0.22 to 0.41	< 0.001
Nutrition strategies → PWBRMS	0.39	0.05	0.30 to 0.49	< 0.001

Panel B. SEM structural model	Standardized *β*	SE	95% CI	*P*
Music engagement → PWBRMS	0.31	0.05	0.21 to 0.41	< 0.001
Nutrition strategies → PWBRMS	0.39	0.05	0.29 to 0.49	< 0.001
Music engagement ↔ nutrition strategies	0.36	0.04	0.28 to 0.44	< 0.001

Panel C. SEM model fit	Value
*χ* ^2^	276.34
Df	150
*χ*^2^/df	1.84
CFI	0.958
TLI	0.949
RMSEA	0.049
90% CI RMSEA	0.039 to 0.058
SRMR	0.041
*R*^2^ (PWBRMS)	0.48

PWBRMS, psychological well-being and recovery-related mental state composite. All covariates were retained in the regression model regardless of significance. SEM fit indices indicate excellent model fit (CFI and TLI > 0.95; RMSEA < 0.05; SRMR < 0.05). *R*^2^ = 0.48 indicates that the model accounts for 48% of variance in PWBRMS.

**Figure 7:**
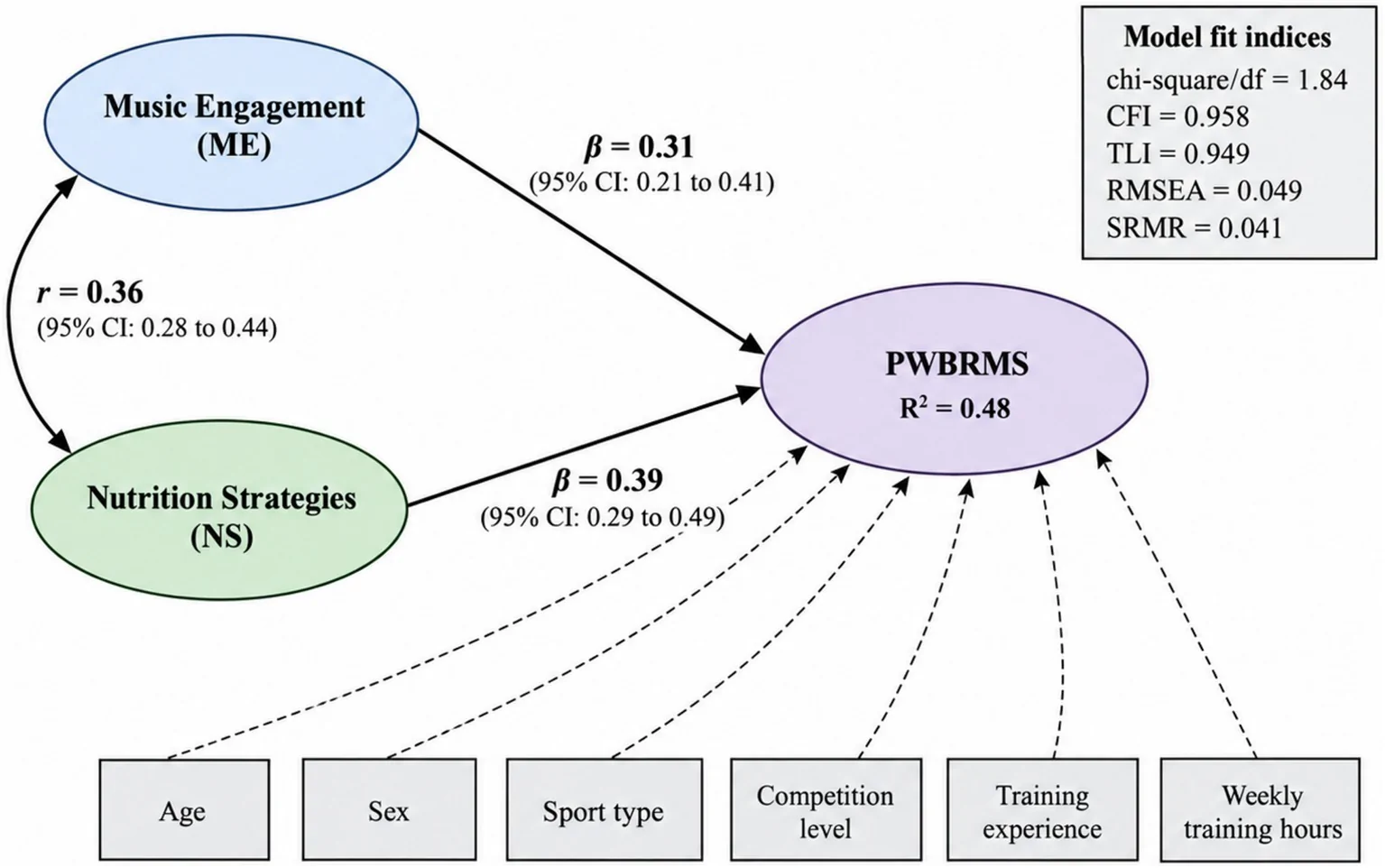
Structural equation model of associations among music engagement (ME), nutrition strategies (NS), and the psychological well-being and recovery-related mental state composite (PWBRMS). Standardized path coefficients are displayed on directional paths. The double-headed arrow represents the freely estimated covariance between ME and NS. Dashed paths represent demographic and training covariates. All paths are cross-sectional associations and do not imply causal direction.

Music engagement was positively associated with the PWBRMS composite (*β* = 0.31, 95% CI 0.22 to 0.41, *p* < 0.001). The substantive meaning of this finding is that athletes who more deliberately and purposefully use music for psychological preparation—selecting playlists for arousal regulation, motivational focus, and emotional recovery—tend to report more favorable psychological functioning across stress, mood, fatigue, readiness, and recovery perception domains. The magnitude of this association (*β* = 0.31) is meaningful in the context of a behavioral correlate collected from a naturalistic sport setting without any experimental manipulation, and it remained stable across regression and SEM estimation, indicating that the association was not an artifact of composite-scoring assumptions.

Nutrition strategies showed a somewhat stronger positive association with PWBRMS (*β* = 0.39, 95% CI 0.30 to 0.49, *p* < 0.001). The comparably larger coefficient for nutrition relative to music suggests that the nutritional dimension of athlete behavioral self-management may carry more proximal relevance to psychological functioning than musical self-regulation alone. This interpretation is mechanistically plausible: adequate energy availability, hydration, and recovery nutrition directly sustain the physiological substrates of mood and cognitive functioning, whereas music primarily operates through psychological and affective channels. The two behavioral constructs also shared a moderate covariance (*r* = 0.36), which the SEM model estimated as a latent-level correlation of 0.36 (95% CI 0.28 to 0.44). This shared variance is consistent with the interpretation that both practices reflect a broader athlete self-management disposition rather than two entirely independent behavioral tendencies, and explains why qualitative participants consistently described them as elements of an integrated daily preparation system. The model accounted for 48% of variance in the PWBRMS latent construct (*R*^2^ = 0.48), which is a substantial explained proportion for a cross-sectional behavioral model in sport psychology, indicating that the combined behavioral predictors and covariates collectively capture a meaningful share of the variability in athlete psychological well-being.

Among the demographic covariates, weekly training volume was the only covariate that reached conventional significance in the adjusted regression model (*β* = −0.09, 95% CI − 0.18 to −0.00, *p* = 0.048). The negative direction of this association heavier training loads corresponding to slightly lower PWBRMS scores is interpretable as the cumulative psychological burden of high-volume training, consistent with athlete overtraining and non-functional overreaching research showing that training load increments are associated with mood disturbance, fatigue accumulation, and perceived recovery deficits. Age, sex, sport type, competition level, and training experience showed no significant independent associations with PWBRMS after adjustment, indicating that the music-nutrition pattern was not confounded by demographic differences in this sample. Figure 8, presented below, displays the standardized regression coefficients with 95% confidence intervals for all predictors in the adjusted model.

**Figure 8:**
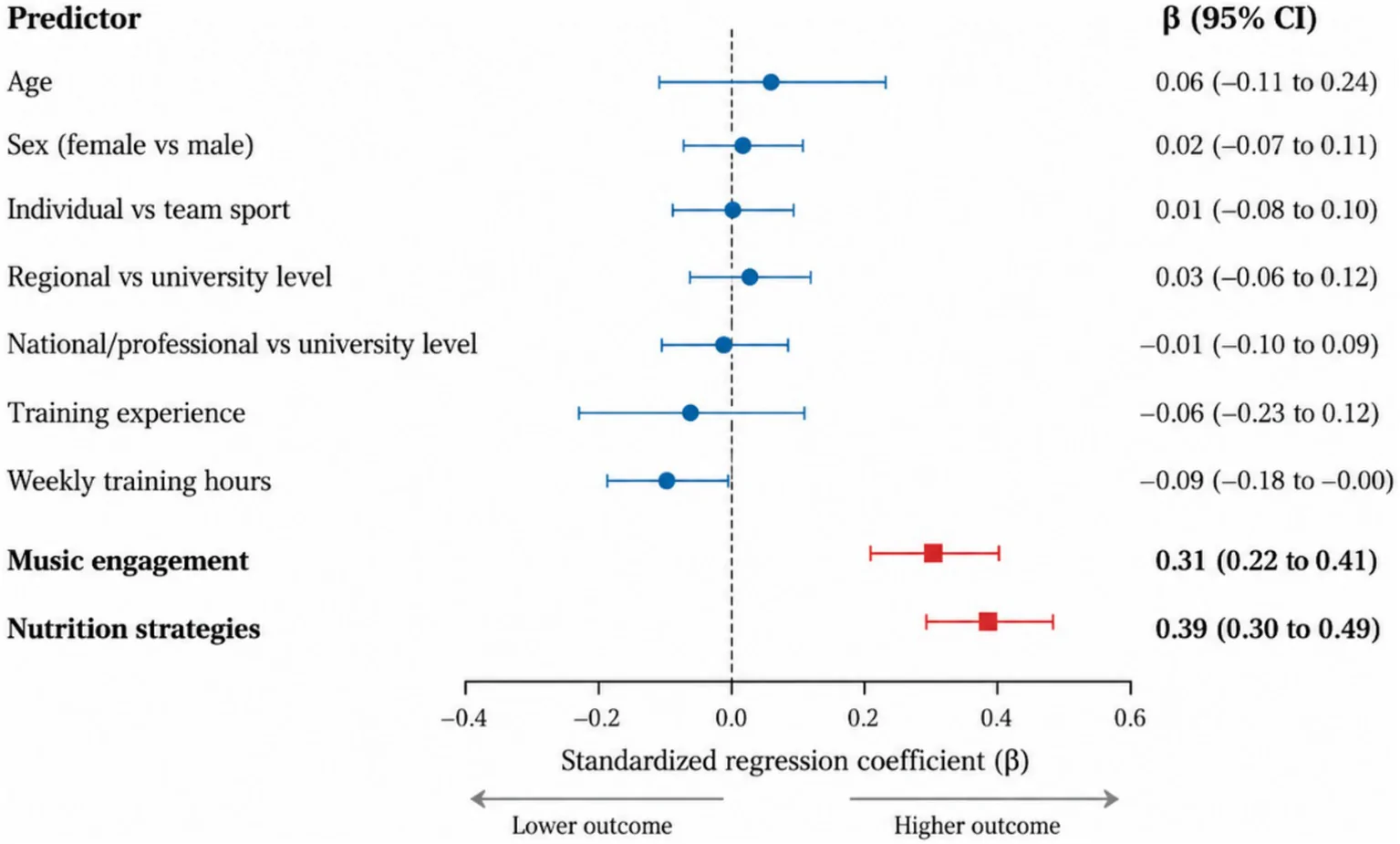
Standardized regression coefficients (*β*) with 95% confidence intervals for all predictors in the adjusted regression model. Coefficients for music engagement and nutrition strategies are highlighted. Covariates with confidence intervals crossing zero were not statistically significant at *α* = 0.05.

### 3.7 Nutrition sub-dimension analysis

To test whether the heterogeneity within the nutrition construct produced meaningfully different associations with the PWBRMS composite, the nutrition strategy measure was decomposed into its two theoretically specified subdimensions. The results, presented in Table 12, indicate that basic nutrition habits (hydration, meal regularity, post-training recovery intake, dietary quality) showed a stronger association with PWBRMS (*β* = 0.30, 95% CI 0.20 to 0.40) than complex nutrition planning behaviors (*β* = 0.21, 95% CI 0.11 to 0.31), both of which remained significant after covariate adjustment. The differential in effect magnitude between the two subdimensions is substantively important. Basic nutrition habits are routinized, low-effort behaviors that can be embedded into existing training structures through environmental cues carrying a water bottle, following team meal schedules and their stronger association with psychological well-being plausibly reflects the cumulative benefits of physiological stability across the training day. When an athlete maintains adequate hydration and consistent energy intake, the physiological substrates underpinning mood, cognitive function (38), and perceived recovery are more consistently supported, producing favorable psychological states. Complex nutrition planning, by contrast, is a more cognitively demanding and resource-intensive behavior. It’s weaker but still significant association with PWBRMS suggests that even imperfect planning selecting appropriate pre-competition meals or adapting intake during travel carries some psychological benefit, potentially through increased perceived control over training demands. Subgroup descriptive patterns by sport type and competition level are visualized in Figure 9.

**Table 12:** Sub-dimension analysis comparing associations of basic nutrition habits and complex nutrition planning with the psychological well-being and recovery-related mental state composite.

Nutrition Sub-dimension	Example behaviors	*β*	SE	95% CI	*P*
Basic nutrition habits	Hydration, meal regularity, recovery intake, dietary quality	0.30	0.05	0.20 to 0.40	< 0.001
Complex nutrition planning	Pre-competition planning, advance meal prep, travel nutrition adjustment	0.21	0.05	0.11 to 0.31	< 0.001

Basic nutrition habits showed a stronger association with PWBRMS than complex nutrition planning. Both subdimensions remained significant after covariate adjustment. The magnitude difference is consistent with the greater feasibility and automaticity of routine nutritional behaviors relative to planning-intensive behaviors.

**Figure 9:**
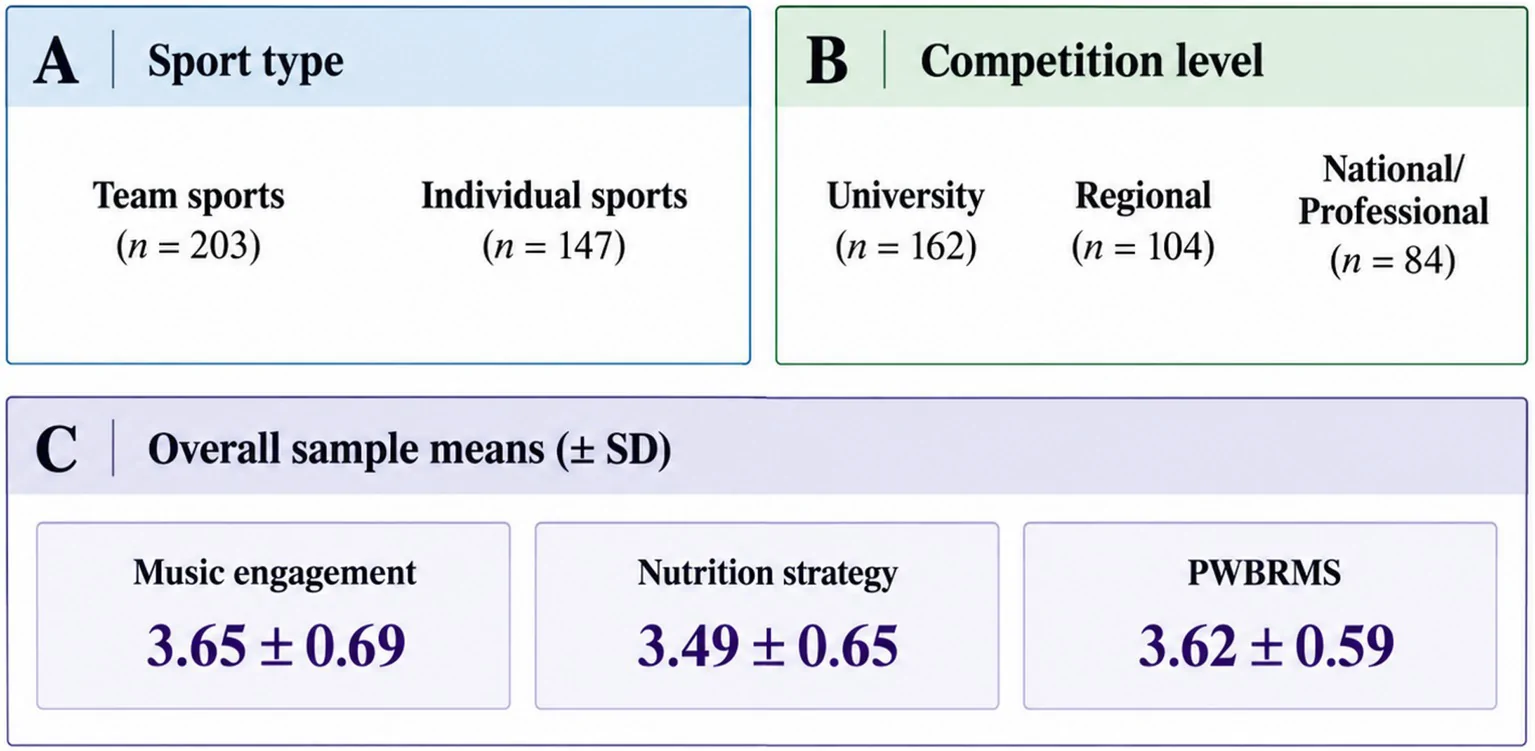
Descriptive patterns in **(A)** sport type, **(B)** competition level, and **(C)** overall mean scores for music engagement, nutrition strategies, and PWBRMS. Error bars represent 95% confidence intervals; subgroup differences are descriptive only.

### 3.8 Qualitative findings and mixed-methods integration

Five themes were identified through codebook thematic analysis of the 30 semi-structured interviews. Rather than repeating each theme in isolation, the qualitative findings are presented here in integrated relation to the quantitative patterns they explain.

The first two themes addressed music engagement. Athletes consistently described music use as a deliberate psychological preparation tool rather than passive background listening, with most selecting specific playlists to create predictable pre-performance routines. One team-sport athlete explained:

"Before competition, I usually listen to the same playlist because it helps me calm down and focus on what I need to do. It is not therapy, but it helps me control my mood and prepare mentally." (Participant 07)

This account contextualizes why the quantitative association between music engagement and PWBRMS (*β* = 0.31) was observed specifically through a strategic-engagement measure rather than a passive-listening scale. Athletes who scored higher on music engagement were those who used music with explicit psychological intent, and it is the intentionality of this use not mere exposure to music that is theoretically relevant to psychological functioning. Divergent accounts were also present: some athletes noted that music became distracting or unhelpful in high-concentration individual sports, cautioning against treating music engagement as universally beneficial and supporting the interpretation that the quantitative association reflects average tendencies rather than uniform effects (39).

The next two themes addressed nutrition strategy heterogeneity. Participants clearly differentiated the effort demands of hydration and meal regularity from those of diet planning and travel nutrition. Hydration was described as structurally embedded into training through environmental cues and team routines, whereas diet planning was characterized as demanding advance planning, financial investment, and scheduling flexibility that many athletes found difficult to sustain. This account:

“Drinking water is easy because I keep a bottle with me during training, but planning meals is harder. When we travel or have late practice, I just eat whatever is available.” (Participant 14).

Directly contextualizes the quantitative pattern in Table 12, where basic nutrition habits (*β* = 0.30) showed a stronger association with PWBRMS than complex nutrition planning (*β* = 0.21). The lower score for diet planning (*M* = 3.28 versus hydration *M* = 3.82) reflects not a lack of nutritional motivation but the structural and logistical constraints that impede planning-intensive behaviors in competitive sport contexts. A third participant quotation speaks to the importance of regular intake for psychological functioning:

“When I eat regularly after training and stay hydrated, I feel less tired and less irritated. If I skip meals, my mood changes and I cannot concentrate well.” (Participant 21).

The fifth theme integrated self-management provides the qualitative explanation for the observed correlation between music engagement and nutrition strategies (*r* = 0.36). Athletes did not describe these practices as independent behavioral choices but as mutually reinforcing components of a unified preparation system encompassing music, nutrition, sleep, and training. This dispositional integration, captured in the statement “For me, music, food, sleep, and training are all connected. If one part is missing, I feel less ready” (Participant 03), suggests that the latent covariance between music engagement and nutrition in the SEM model reflects a genuine shared behavioral tendency rather than statistical artifact. The integrated joint display in Table 4 synthesizes these quantitative-qualitative correspondences, and Figure 10 presents the non-directional thematic map of athlete self-management practices.

**Figure 10:**
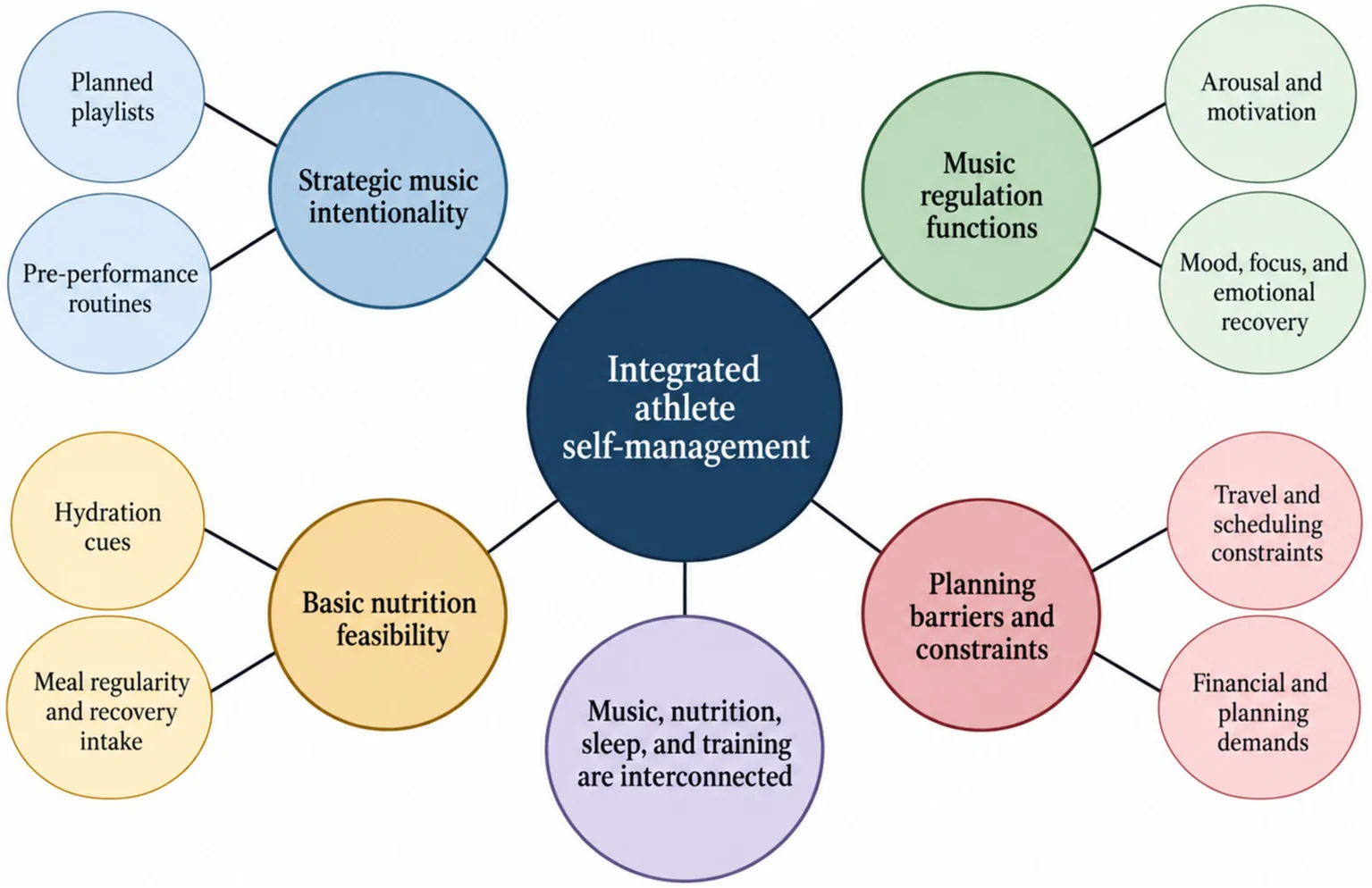
Non-directional thematic map derived from qualitative codebook thematic analysis. Nodes represent themes and subthemes. Connections indicate thematic relationships identified in participant accounts. Arrow direction is not shown because the thematic analysis does not establish causal or temporal ordering among themes.

## 4 Discussion

This sequential explanatory mixed-methods study examined the cross-sectional associations of strategic music engagement and differentiated nutrition strategies with a psychological well-being and recovery-related mental state composite among 350 competitive athletes, and used qualitative interviews to provide explanatory context for those associations (40). The principal quantitative findings that both behavioral practices showed positive, moderate-magnitude associations with athlete-reported psychological functioning, that nutrition strategies showed a comparably stronger association than music engagement, and that basic nutrition habits outperformed complex nutrition planning were interpreted through qualitative accounts describing the intentionality of music use, the effort-differential within nutrition behaviors, and the integrative self-management logic with which athletes organized these practices in everyday sport life (41). Together these findings advance the understanding of non-clinical, athlete-centered psychological self-management in several directions.

The positive association between strategic music engagement and the PWBRMS composite (*β* = 0.31) confirms and extends the existing literature on music in sport and exercise. Meta-analytic work by Terry and colleagues established that music can influence affective valence, motivation, and perceived exertion during exercise, with effect sizes moderated by the degree of intentional music selection. The present finding adds to this evidence base by demonstrating that strategic music engagement assessed in a naturalistic competitive sport context rather than an experimental laboratory is associated with a multidimensional psychological functioning composite that extends beyond single-item affect or arousal measures (42). Importantly, the association magnitude was consistent across observed-score regression and latent-variable SEM estimation, which strengthens confidence that the finding is not an artifact of the analytical representation chosen. The qualitative accounts further reveal that the relevant mechanism is likely attitudinal and regulatory: athletes who use music purposefully create predictable psychological transitions between daily training demands and competition preparation states, a function that aligns with what attentional resource theories predict about the capacity of music to occupy cognitive space and buffer negative affective intrusions (43). This interpretation extends beyond purely arousal-based explanations of music in sport and positions strategic music use as a form of proactive emotion regulation consistent with self-determination theory frameworks, in which autonomous and intentional behavioral regulation supports psychological well-being (44).

The somewhat stronger association between nutrition strategies and PWBRMS (*β* = 0.39) relative to music engagement is both theoretically expected and practically meaningful. Nutritional psychiatry research linking dietary quality and dietary improvement to reduced depressive and anxiety symptoms in non-clinical populations provides a plausible conceptual basis for this association, though that literature is predominantly drawn from general community samples rather than competitive athlete populations. In athlete-specific contexts, sports nutrition position statements articulate energy availability, carbohydrate and protein periodization, hydration, and recovery nutrition as core determinants of training adaptation and readiness, and the present findings suggest that their relevance extends into the psychological domain as well (45). The biological plausibility rests on several mechanisms: adequate energy intake prevents the physiological and neuroendocrine disruptions associated with low energy availability and relative energy deficiency in sport (RED-S), which include mood dysregulation, cognitive impairment, and fatigue amplification; hydration sustains cerebral perfusion and neurotransmitter balance, influencing cognitive function and emotional valence even at mild deficit levels; and recovery nutrition practices that replenish glycogen and support protein synthesis reduce the accumulating physiological stress that athletes experience as mood disturbance and readiness impairment (46). Together these pathways explain why nutrition strategies showed a somewhat stronger statistical association with psychological functioning than music engagement, whose primary mechanisms of action are psychological and affective rather than physiological.

The subdimension analysis revealing that basic nutrition habits (*β* = 0.30) showed a stronger association with PWBRMS than complex nutrition planning (*β* = 0.21) is a finding that prior research has rarely been positioned to detect, precisely because most existing nutrition studies treat dietary behavior as a single composite score (47). The differential is theoretically meaningful because it suggests that the psychological benefits of athlete nutrition are more reliably connected to consistent, routinized fueling behaviors hydration, regular meals, post-training recovery intake (48) than to higher-order planning behaviors that require executive function, advance scheduling, and resource access. This pattern converges with habit formation and behavioral automaticity frameworks, which propose that behaviors embedded into existing routines through reliable environmental cues require less cognitive effort to maintain and therefore generate more stable behavioral patterns that can support consistent physiological and psychological outcomes (49). The qualitative accounts reinforced this interpretation: athletes described hydration as embedded into training through cue-dependent habit structures, whereas diet planning broke down under the pressure of travel schedules, financial constraints, and irregular competition demands. The practical implication for athlete support services is that interventions should prioritize habit-stacking of high-frequency basic nutritional behaviors before attempting to impose complex planning requirements on athletes whose competitive environments make such planning difficult to sustain (50).

The moderate covariance between music engagement and nutrition strategies (*r* = 0.36) presents an interesting interpretive challenge. One explanation is statistical: both behavioral scales were drawn from the same self-report questionnaire, and common rater tendencies could inflate their apparent correlation (51). The CMV diagnostics, however, suggest that the covariance is not fully explained by shared method variance, with average method-related variance estimated at 11.6% and structural path estimates changing by at most 0.04 after LCMF inclusion. A more substantive explanation, supported by the qualitative fifth theme, is that athletes who use music strategically and maintain nutritional routines share a common dispositional orientation toward deliberate psychological self-management (52). Qualitative participants explicitly described music and nutrition as elements of the same preparation system, embedded alongside sleep routines and training structure into a coherent pre-performance behavioral pattern. This dispositional explanation aligns with self-regulation theories that identify individual differences in the tendency to implement goal-directed behavioral routines as a stable trait-like orientation, and it suggests that intervention approaches targeting both behavioral domains together rather than individually may be more effective for athletes whose self-management orientation is already well-developed (53).

The present study extends previous mixed-methods work in sport psychology by demonstrating that the sequential explanatory design adds genuine interpretive value beyond what either strand alone would provide. The quantitative models identified the presence and magnitude of behavioral associations with psychological functioning, while the qualitative themes identified the mechanisms intentionality of music selection, behavioral feasibility of nutritional routines, contextual barriers to planning, and integrative self-management logic that explain why those associations exist (54). This explanatory function is precisely the contribution that sequential explanatory designs are theoretically intended to offer, and its realization in the present study strengthens the case for mixed-methods integration as a methodological standard in athlete behavioral research (55). The common method variance diagnostics reported here a three-pronged strategy rarely implemented comprehensively in athlete survey research (56) further enhance the credibility of the quantitative findings by demonstrating that they are unlikely to be fully attributable to shared measurement context.

Several considerations qualify the interpretation of these findings (57). The cross-sectional design does not permit causal inference, and the directionality of the observed associations is indeterminate from these data alone. It is equally plausible that athletes experiencing more favorable psychological states are better positioned to implement consistent nutritional routines and deliberate music preparation strategies—a reverse-causation pathway that longitudinal or experimental designs would be needed to distinguish (58). The sample comprised predominantly younger university-level athletes with a mean age of approximately 23 years, and the patterns may not generalize to older professional athletes who operate in markedly different training environments with greater institutional nutrition support and more established pre-competition routines (59). The study-specific measurement instruments, while demonstrating acceptable psychometric properties within this sample, require independent validation in diverse athlete populations before their measurement equivalence can be assumed (60). These qualifications do not undermine the substantive significance of the findings but underscore that the present results should be treated as hypothesis-generating observations that motivate prospective and experimental follow-up.

## 5 Conclusion

This sequential explanatory mixed-methods study demonstrated that strategic music engagement and differentiated nutrition strategies are independently and positively associated with athlete-reported psychological well-being and recovery-related mental state among competitive athletes. Music engagement showed a moderate positive association (*β* = 0.31) and nutrition strategies a comparably stronger one (*β* = 0.39), together accounting for 48% of variance in the psychological functioning composite after adjustment for demographic and training covariates. Within the nutrition domain, basic nutrition habits showed a stronger association with psychological well-being than complex nutrition planning, a pattern explained by the greater feasibility and behavioral automaticity of routine fueling behaviors relative to planning-intensive practices. A moderate covariance between music engagement and nutrition strategies (*r* = 0.36) suggests that both practices reflect a shared dispositional orientation toward deliberate self-management, which qualitative participants consistently described as an integrated daily preparation system encompassing music, nutrition, sleep, and training.

These findings advance athlete psychological health research by providing the first simultaneous, measurement-validated examination of strategic music use and differentiated nutrition strategies as behavioral correlates of psychological functioning in a competitive sport sample, using a design that integrates rigorous quantitative modeling with qualitative explanatory depth. The results support the relevance of non-clinical, athlete-centered behavioral self-management for psychological well-being without overstating causal claims that the observational design cannot sustain. For sport psychologists, coaches, and sports nutrition professionals, the findings suggest that supporting athletes in developing consistent nutritional routines and purposeful music-use habits may complement existing psychological support frameworks, particularly at university and regional competitive levels where institutional support is less intensive. These associations should be treated as hypothesis-generating, and their causal interpretation awaits longitudinal and intervention-based investigation.

### 5.1 Limitations and future research directions

Several methodological constraints qualify the interpretation of these findings. The cross-sectional observational design prevents causal inference and temporal ordering, leaving open the possibility that athletes with more favorable baseline psychological states are better positioned to sustain consistent nutritional routines and deliberate music preparation strategies. Longitudinal diary studies and randomized behavioral trials are needed to establish directionality and test whether changes in these practices produce corresponding changes in psychological functioning. The sample comprised predominantly younger university-level athletes (mean age 22.85 years) from a single Chinese city, which limits generalizability to older professional athletes, diverse cultural settings, and competitive environments with markedly different institutional support structures. The study-specific music engagement and nutrition strategy instruments demonstrated acceptable preliminary psychometric properties within this sample but require independent validation across diverse athlete populations, languages, and competitive levels before measurement equivalence can be assumed. The self-report measurement of all constructs at a single time point, despite the CMV diagnostics applied, cannot fully exclude residual common method bias. Future research should incorporate objective behavioral indicators of nutrition and music use alongside validated clinical and athlete-specific mental health instruments to more precisely separate psychological well-being from training load and recovery status, and to evaluate whether the associations reported here replicate under more controlled measurement conditions.

## Data Availability

The raw data supporting the conclusions of this article will be made available by the authors, without undue reservation.
